# Hydrogen Sulfide Modulates Adult and Reparative Neurogenesis in the Cerebellum of Juvenile Masu Salmon, *Oncorhynchus masou*

**DOI:** 10.3390/ijms21249638

**Published:** 2020-12-17

**Authors:** Evgeniya V. Pushchina, Maria E. Stukaneva, Anatoly A. Varaksin

**Affiliations:** Zhirmunsky National Scientific Center of Marine Biology, Far Eastern Branch, Russian Academy of Sciences, 690041 Vladivostok, Russia; Stykanyova@mail.ru (M.E.S.); anvaraksin@mail.ru (A.A.V.)

**Keywords:** traumatic brain injury, adult neuronal stem cells, neuronal progenitor cells, nestin, vimentin, H_2_S, glutamine synthetase, cerebellum, masu salmon, adult neurogenesis

## Abstract

Fish are a convenient model for the study of reparative and post-traumatic processes of central nervous system (CNS) recovery, because the formation of new cells in their CNS continues throughout life. After a traumatic injury to the cerebellum of juvenile masu salmon, *Oncorhynchus masou*, the cell composition of the neurogenic zones containing neural stem cells (NSCs)/neural progenitor cells (NPCs) in the acute period (two days post-injury) changes. The presence of neuroepithelial (NE) and radial glial (RG) neuronal precursors located in the dorsal, lateral, and basal zones of the cerebellar body was shown by the immunohistochemical (IHC) labeling of glutamine synthetase (GS). Progenitors of both types are sources of neurons in the cerebellum of juvenile *O. masou* during constitutive growth, thus, playing an important role in CNS homeostasis and neuronal plasticity during ontogenesis. Precursors with the RG phenotype were found in the same regions of the molecular layer as part of heterogeneous constitutive neurogenic niches. The presence of neuroepithelial and radial glia GS+ cells indicates a certain proportion of embryonic and adult progenitors and, obviously, different contributions of these cells to constitutive and reparative neurogenesis in the acute post-traumatic period. Expression of nestin and vimentin was revealed in neuroepithelial cerebellar progenitors of juvenile *O. masou*. Patterns of granular expression of these markers were found in neurogenic niches and adjacent areas, which probably indicates the neurotrophic and proneurogenic effects of vimentin and nestin in constitutive and post-traumatic neurogenesis and a high level of constructive metabolism. No expression of vimentin and nestin was detected in the cerebellar RG of juvenile *O. masou*. Thus, the molecular markers of NSCs/NPCs in the cerebellum of juvenile *O. masou* are as follows: vimentin, nestin, and glutamine synthetase label NE cells in intact animals and in the post-traumatic period, while GS expression is present in the RG of intact animals and decreases in the acute post-traumatic period. A study of distribution of cystathionine β-synthase (CBS) in the cerebellum of intact young *O. masou* showed the expression of the marker mainly in type 1 cells, corresponding to NSCs/NCPs for other molecular markers. In the post-traumatic period, the number of CBS+ cells sharply increased, which indicates the involvement of H_2_S in the post-traumatic response. Induction of CBS in type 3 cells indicates the involvement of H_2_S in the metabolism of extracellular glutamate in the cerebellum, a decrease in the production of reactive oxygen species, and also arrest of the oxidative stress development, a weakening of the toxic effects of glutamate, and a reduction in excitotoxicity. The obtained results allow us to consider H_2_S as a biologically active substance, the numerous known effects of which can be supplemented by participation in the processes of constitutive neurogenesis and neuronal regeneration.

## 1. Introduction

Fish are a convenient model for studying the reparative and post-traumatic central nervous system (CNS recovery), because the formation of new cells in their CNS continues throughout their life [1]. The pattern of distribution of proliferative centers in the teleost fish brain is strikingly similar, despite significant differences in the ecological specialization and phylogenetic remoteness between fish species [2]. *Danio rerio* showed the presence of 16 neurogenic zones located along the rostro-caudal axis of the brain [3,4,5]. In addition to the widespread cell proliferation and the ability to create a large number of new cells in CNS, the teleost fish brain can successfully recover after being damaged [6,7,8,9,10].

Salmonids, which are a phylogenetically ancient group, have a high concentration of undifferentiated elements, both in the matrix zones of the brain and in the parenchyma [11]. Their ontogenesis is characterized by such phenomena as developmental delay and retention of signs of the embryonic organization of CNS which occur at the stage of active brain growth, where the morphogenesis processes are most clearly and fully expressed [11]. This feature of salmon CNS development, referred to as embrionalization, is confirmed by the presence of a large number of embryonic neuronal stem cells (NSCs) corresponding to cells of the neuroepithelial (NE) type, as well as radial glial (RG) cells corresponding to adult progenitors [12,13,14,15].

It has been established that the proportion of NE and RG precursors varies in different periods of postembryonic neurogenesis in fish [16]. During embryogenesis and in the postembryonic period, a heterogeneous population of NSCs/neuronal progenitor cells (NPCs) includes NE, RG, and other types of progenitors that together form neurons, glial, and ependymal cells constituting the basis of the CNS [17,18]. Such cells differ in the composition of expressed molecular markers characterizing their phenotype and ability to proliferate or dormancy [19]. It has been found that NE cells have a high potential as NSCs; they originate from embryonic NSCs in the early stages of CNS ontogenesis and are characterized by high multipotency, as well as become various types of neurons and a heterogeneous population of glial cells [16]. An essential feature of the organization of glial cells is their polarization along the apical–basal axis and, in some cases, the presence of a cilium on the apical domain of a neuroepithelial cell [20]. NE cells are self-renewing and multipotent; they create most of the cells in the CNS [16]. NE cells are a predominant population of NPCs, which is maintained in the cerebellum of *D. rerio* throughout life [4,21,22].

Glutamine synthetase (GS) is a molecular marker of NSCs and is detected in RG cells in the brain of adult amphibians [23], teleost [24], and cartilaginous fish [25]. However, the data on glutamine synthetase labeling of NE and RG cells in the cerebellum of *D. rerio* [21] and *Apteronotus leptorhynchus* [1,26,27] differ substantially. This indicates a heterogeneous population of cells labeled with GS in different parts of the brain, as well as interspecies differences. The mechanisms of constitutive neurogenesis in the fish brain, as well as the cellular composition of neurogenic niches in various parts of the fish brain, including the matrix zones of the cerebellum, are currently investigated [21]. The involvement of GS+ cells in various parts of the brain in the post-traumatic response still remains unclear.

Vimentin (Vim) and nestin (Nes) are currently also considered as markers of NSCs. It has been shown that in the proliferative zones of the telencephalon of juvenile *O. masou* [15] and the mesencephalic tegmentum of juvenile chum salmon *O. keta* [14], vimentin is present in NE cells and is not detected in the RG of intact animals. Studies of the immunolabeling of nestin in the tegmentum of juvenile *O. keta* have also shown its presence in NE cells located both in the periventricular zone (PVZ) and in the tegmental parenchyma, as well as the presence of widespread Nes+ granules in the mesencephalic reticular formation in intact juvenile chum salmon [14]. After a traumatic brain injury (TBI), Vim+ RG appears in the telencephalon of juvenile *O. masou* and *O. keta* and is absent in intact animals [14]. Studies on *D. rerio* have shown Vim expression predominantly in neurons at the initial stage of differentiation and, possibly, in glial cells found in various brain regions [28]. Studies on grey mullet *Chelon labrosus* have shown a close relationship between the expression of vimentin and glial fibrillar acidic protein (GFAP), whose proportion changes during ontogenesis [29]. During the CNS development, vimentin is replaced by GFAP in reptiles [30], birds [31], and mammals [32].

Nestin is also expressed by NPCs in vivo and in vitro in the subventricular zone of the lateral ventricle and the subgranular zone of the *dentate gyrus* in the hippocampus of adult brain [3,33,34,35,36,37,38,39,40]. Nestin is one of the RG molecular markers [41] and is almost not expressed by mature neurons [34]. In differentiated cells, the expression of nestin is replaced by the expression of proteins specific for neurons or glia [33]. In *D. rerio*, as in mice, the nestin expression is retained in neurogenic niches containing NSCs and proliferating NPCs [3,42,43,44]. Studies on the localization of nestin after a tegmentum injury in juvenile chum salmon showed a significant increase in Nes+ NE cells that form local post-traumatic foci, as well as in RG fibers, which were not detected in intact juveniles [14]. After a TBI of the tegmentum in juvenile *O. keta*, patterns of granular localization of nestin, which form zones adjacent to the damaged area, were also revealed [14]. Thus, taking into account the heterogeneous composition of constitutive and reactive neurogenic zones in the tegmentum of juvenile salmonids, as well as the heterogeneous pattern of NSCs/NPCs labeling using nestin and vimentin, one of the objectives of this study was to determine the molecular phenotypes of NSCs/NPCs using these two markers in the cerebellum of juvenile *O. masou*.

H_2_S plays an essential role in post-embryonic neurogenesis and in post-traumatic neuronal regeneration in adult animals by activating certain molecular pathways and cell–cell interactions [12,45]. H_2_S, like other gaseous transmitters, is spread by diffusion, which determines its involvement in the processes of intercellular signaling such as, in particular, the regulation of calcium fluxes in cell–cell interaction [46]. H_2_S in the brain is synthesized by enzymatic and non-enzymatic pathways [45,47] and has a great influence on physiological and pathophysiological processes in mammal CNS, participating in defense mechanisms after TBI [47]. Taking into account the fact that the inflammatory response arising in fish after TBI promotes neuronal regeneration and prevents the formation of the glial scar [8], H_2_S can be considered as one of the factors contributing to successful post-traumatic regeneration and neuroprotection in the fish brain. To test this hypothesis, we studied the features of expression of molecular markers of NSCs/NPCs: GS, nestin, vimentin. CBS is measured as indicative of H_2_S presence in the cerebellum, being the enzyme that catalyzes the reaction producing H_2_S from L-Cysteinein in the cerebellum of intact juvenile *O. masou*, as well as in fish during the acute post-traumatic period.

## 2. Material and Methods

### 2.1. Experimental Animals

The study was carried out on 40 one-year-old juveniles of the masu salmon *O. masou* with a body length of 9–11.5 cm and a weight of 20–35 g. The animals were obtained from the Ryazanovka experimental fish hatchery in 2018. The juveniles were kept in a tank with aerated fresh water at a temperature 16–17 °C and fed once a day. The daily light/dark cycle was 14/10 h. The concentration of dissolved oxygen in the water was 7–10 mg/dm^3^, which corresponds to normal saturation. All experimental manipulations with animals were in accordance with the rules listed in the charter of the A.V. Zhirmunsky National Scientific Center of Marine Biology (NSCMB) FEB RAS and the Ethical Commission regulating the humane treatment of experimental animals (approval # 3-061020 from Meeting No. 3 of the Commission on the biomedical ethics of NSCMB FEB RAS, October 6, 2020). The animals were divided into two groups. The animals in the control group were intact (*n* = 20); the experimental groups consisted of fish with damaged cerebellum (*n* = 20).

### 2.2. Experimental Injury of the Cerebellum

The animals were caught with a net from the aquarium in which they were kept and quickly inflicted injury in accordance with the method of Zupank and Ott [48]: a 1 mm deep wound was made with a thin sterile needle driven into the dorsal region of the cerebellar body. The area of injury covered both the molecular and granular layers of the cerebellar body and did not affect the other parts of the brain. Immediately after the TBI, the animals were released back into the tank with fresh water for recovery and further monitoring. Video monitoring of changes in motor and behavioral activity of fish was conducted during the first hour after the injury. In the animals with the cerebellar injury, the motor activity significantly increased compared with the control group: they jumped out of the water and performed rotational movements [49]. These observations indicate a damage of the structure of the cerebellum and downstream motor centers of the reticular formation. A small hematoma of 1–2 mm in size was clearly visible in the area of injury.

### 2.3. Preparation of Material for Immunohistochemical Studies

Anesthesia and prefixation. At 2 days after the TBI, the animals were removed from the experiment and euthanized by the method of rapid decapitation. The fish were anesthetized in a 0.1% solution of ethyl-3-aminobenzoate methanesulfonate (MS222) (Sigma, Cat. # WXBC9102V, St. Louis, MO, USA) for 10–15 min. After the decapitation, the intracranial cavity of the animals was prefixed with a 4% paraformaldehyde solution prepared in 0.1 M phosphate buffer (pH 7.2). After the prefixation, the brain was removed from the cranial cavity and fixed in the 4% paraformaldehyde solution for 2 h at 4 °C. Then they were kept in a 30% sucrose solution at 4 °C for two days (with the solution changed seven times). Serial frontal brain sections with a thickness of 50 μm were made on a freezing microtome (Cryo-star HM 560 MV, Oberkochen, Germany). Every third frontal section of the cerebellum was taken for the reaction.

### 2.4. Immunohistochemical Verification of Vimentin, Nestin, Glutamine Synthetase, and Cystathionine β-Synthase (CBS NE 4.2.1.22)

To study the expression of nestin, vimentin, glutamine synthetase and cystathionine β-synthase in the cerebellum of juvenile *O. masou*, immunoperoxidase labeling was performed on frozen, free floating brain sections. Monoclonal mouse antibodies against GS (catalog number GF5 ab10062), vimentin (catalog number 3B4 ab28028), nestin (catalog number 2C1.3A11 ab18102), and CBS (catalog number, ab54883) from Abcam (Cambridge CB2 0AX, UK) at a dilution of 1:300 were used on frontal 50 μm sections incubated in situ at 4 °C for 48 h. Protein expression was assessed at 2 days after the TBI to the cerebellum. To visualize immunohistochemical (IHC) labeling, the ready-to-use biotinylated secondary horse antibody and standard ABC (avidin biotin complex) (Vectastain Elite ABC kit; Cat. no. 6100; Vector Laboratories, San Francisco, CA, USA) was used in accordance with the manufacturer’s recommendations. To identify the reaction products, a red substrate (VIP Substrate Kit, catalog number SK-4600, Vector Laboratories, Burlingame, CA, USA) was used in accordance with the manufacturer’s recommendations. The brain sections were placed on polylysine-coated glass slides (BioVitrum, St. Petersburg, Russia) and left to dry completely. For identification of immune-negative cells, the cerebellum sections were additionally stained with a 0.1% methyl green solution (Bioenno, Lifescience, CA, USA, Cat # 003027). The color development was monitored under a microscope. The sections were washed in three changes of distilled water for 10 s, differentiated for 1–2 min in a 70% alcohol solution, and then for 10 s in 96% ethanol. The brain sections were dehydrated in accordance with the standard procedure: placed in two changes of xylene, 15 min each, and then embedded in the Bio-optica medium (Milano, Italy) under coverslips.

The negative control method was used to assess the specificity of the IHC reaction. The brain sections, instead of primary antibodies, were incubated with a 1% solution of non-immune horse serum for 1 day and then processed as sections with primary antibodies. In all control experiments, there was no immunopositive reaction.

### 2.5. Microscopy

To visualize cell bodies and carry out a morphological and morphometric analysis of their parameters (measurements of the greater and lesser diameters of the soma), a Zeiss Axiovert 200M fluorescence motorized phase contrast microscope with an ApoTome fluorescence module and AxioCam MRM and AxioCam HRC digital cameras (Carl Zeiss, Jena, Germany) was used. The material was analyzed using the AxioVision software. Measurements were performed at 100×, 200×, 400× magnifications and in several randomly selected fields of view for each study area. The number of labeled cells in the field of view was counted at a magnification of 200×. Micrographs of the sections were taken with an Axiovert 200 digital camera. The material was processed using the Axioimager program and the Corel Photo-Paint 12 graphics editor. The optical density (OD) of IHC labeling products in neuronal bodies and immunopositive granules was measured using the Axiovert 200-M microscope software. For this purpose, the Wizard program conducted a standard assessment of optical density for 5–7 sections, choosing 10–15 intensively/moderately labeled and immunonegative cells of the same type for analysis. Then, the average value of optical density for each type of cells was subtracted from the maximum value of optical density for immunonegative cells (background), and the actual values were expressed in relative units of optical density (UOD).

### 2.6. Stereological Method in the Study of Quantitative Parameters of the Cerebellum

To obtain reliable quantitative characteristics of various regions of the cerebellum of juvenile *O. masou*, as a volumetric object in space, we used the stereological method of calculating the data obtained during microscopic analysis. For reliable spatial reconstruction for ICH study, we used every 3 sections of the cerebellum. The stereological method enables, with the reliability determined by the objectives of the study and controlled by varying the parameters of the study design, the revealing of the morphometric characteristics of the object under study on the material of a limited number of sections. In this case, the systematic error (bias) is proven to be avoided, and the measurement error is controllable and directly depends on the sampling frequency: the more slices, the higher the accuracy. This is achieved through the use of an appropriate mathematical apparatus and adherence to sampling rules, in particular, systematic random sampling.

When working with sections of the cerebellum, we selected the area under study, after which we took into account the morphometric parameters of interest. After receiving data from all selected slices, we performed calculations that allowed us to proceed to the description of the volumetric object. In particular, data were obtained on parameters such as the number of immunopositive cells within the dorsal, lateral and basal zones of the cerebellum, the density of distribution of immunopositive cells in constitutive and reactive neurogenic niches, and the number of immunopositive cells and granules in the periventricular, subventricular and parenchymal zones of all layers of the cerebellum.

### 2.7. Statistical Analysis

Prior to the experiments, we performed a statistical analysis based on the variations in the measured parameters in our previous research [15] and determined that we needed a group of at least 4 animals to achieve the statistical confidence at 95%. To make sure that we reach a group size of 4 and, at the same time, reduce the use of animals to a minimum, we aimed for a total of 5 animals per experimental group.

All data are representative of at least three independent experiments as means ± standard deviation of the mean (M ± SD) and analyzed using the SPSS software application (version 16.0; SPSS Inc., Chicago, IL, USA). The quantitative processing of morphometric data of IHC labeling was performed using the Statistica 12 and Microsoft Excel 2010 and STATA software packages (StataCorp. 2012, Stata Statistical Software: Release 12. College Station, TX: StataCorp, LP, College Station, TX, USA). Data between control (intact) and experimental (2 days after injury) groups were validated for significance using the Student–Newman–Keuls *t*-test. Comparison of the number of immunopositive cells between several neuroanatomical areas of the intact cerebellum (presented as groups) was performed using one-way analysis of variance (ANOVA, Chicago, IL, USA) with Bonferroni’s correction. Values at *p* ≤ 0.01 and *p* ≤ 0.05 were considered statistically significant. 

## 3. Results

### 3.1. Labeling of Progenitor Cells with Vimentin in the Cerebellum of Intact Juvenile O. masou and on Day 2 Post-Injury

**Figure 1:**
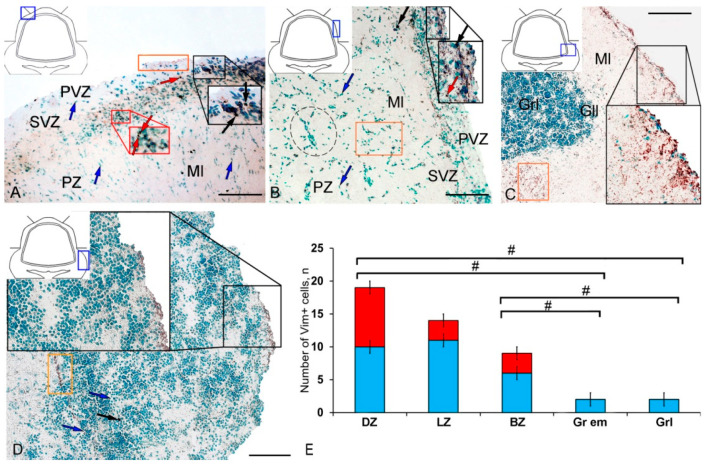
Distribution of vimentin in the intact cerebellum of juvenile masu salmon, *Oncorhynchus masou*. (**A**) Dorsal zone: Vim+ cells 1 type (red arrows); Vim− cells 2 type (black arrows), aggregation of Vim+ cells in the periventricular zone (PVZ) of the molecular layer (black inset); aggregation of Vim+ cells in the subventricular zone (SVZ) of the molecular layer (red inset); areas of extracellular vimentin deposition are outlined by orange rectangle; molecular layer (Ml). (**B**) Lateral zone (see designations in Figure 1A): black dotted lines outline clusters of Vim− cells; subventricular zone (SVZ), parenchymal zone (PZ). (**C**) Basal zone, ganglionic layer (Gll), granular layer (Grl), and other designations as in Figure 1A. (**D**) Granular eminences. (**E**) The comparative distribution of Vim+ cells in different zones of cerebellum in intact fish (*n* = 5 in each group; # are significant intergroup differences) One-way analysis of variance (ANOVA). Immunohistochemical (IHC) labeling of vimentin. Blue rectangle in the pictogram indicates the corresponding area in the micrograph. Scale bars: (**A**–**D**) 100 μm.

In intact animals, the IHC labeling of vimentin was detected in the dorsal zone (DZ), lateral zone (LZ), and basal zone (BZ) of the cerebellar body, and in granular eminences. Due to the vimentin labeling, two types of cells, as well as granules of subcellular size, were identified. The morphological parameters of Vim+ elements are shown in Supplementary Materials Table S1. Small, intensely labeled cells of types 1 and 2 were detected in the periventricular zone (PVZ) of the molecular layer of the DZ (Figure 1A). There were both small clusters containing cells of types 1 and 2, and single cells located in the subventricular zone (SVZ) and parenchymal zone (PZ) (Figure 1A, Table S1). The intensity of labeling of small cells ranged from moderate to high. In the PZ, there were aggregations of moderately or weakly labeled granules of subcellular size, forming small morphogenetic fields (Figure 1A, Table S1). In the LZ, the pattern of distribution of Vim+ cells was slightly different; in the PVZ, aggregations of Vim+ cells and diffuse-type granules prevailed; in the SVZ, there were single or paired, oval type 2 cells (Figure 1B, Table S1). In the PZ, an increased concentration of Vim-negative cells in the state of radial or tangential migration was detected (Figure 1B). A granular pattern of weak Vim+ localization dominated the BZ (Figure 1C, Table S1). In the PVZ and SVZ, the localization of Vim+ cells and granules generally corresponded to that in other zones (Figure 1C). In granular eminences (Gr em), we detected a high concentration of Vim−negative (Vim−) cells, among which there were single intensely labeled Vim+ cells of type 1 and a weakly expressed immunolabeled neuropil in the granular layer (Figure 1D, Table S1).

The results of the quantitative analysis showed that in the intact animals the maximum number of Vim+ cells were in the DZ and gradually decreased in the LZ and BZ (Figure 1E). The minimum number of Vim+ cells was found in Gr em and in the granular layer (Figure 1E).

**Figure 2:**
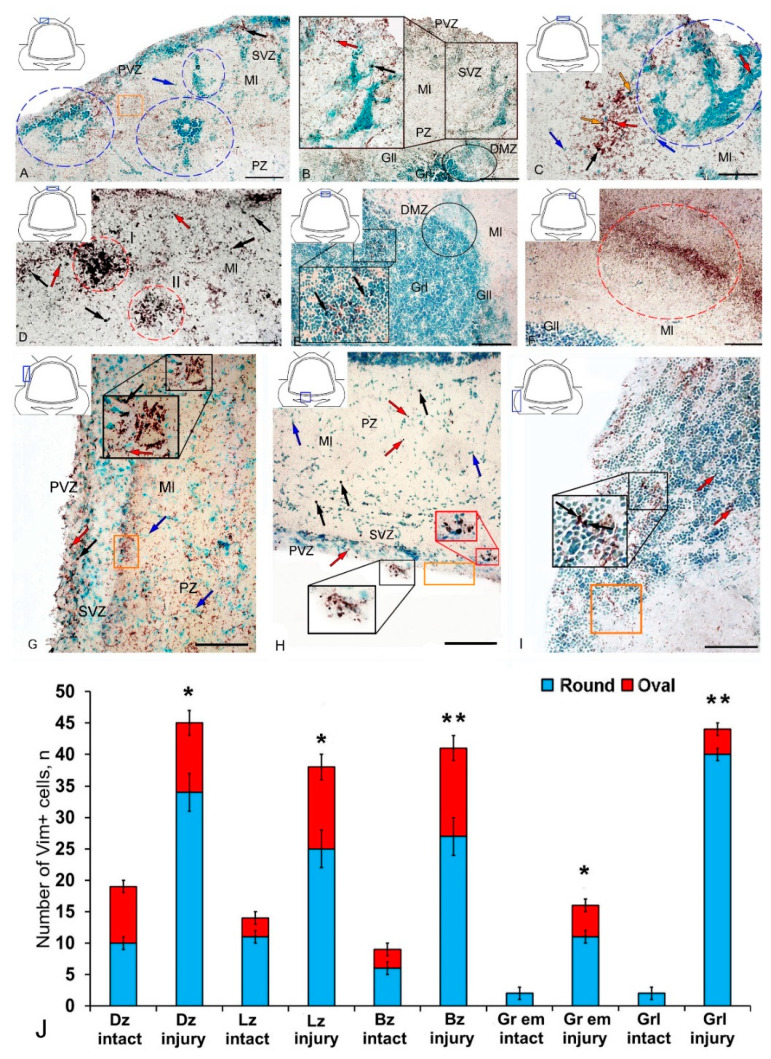
Distribution of vimentin in the cerebellum of juvenile masu salmon *Oncorhynchus masou* on day 2 post-injury. (**A**) Dorsal zone (DZ): Vim+ cells (black arrows); Vim− cells (blue arrows); areas of extracellular deposition of vimentin are outlined by orange rectangle; reactive neurogenic niches are outlined by blue dashed ovals; periventricular zone (PVZ), subventricular zone (SVZ), parenchymal zone (PZ), molecular layer (Ml). (**B**) Enlarged fragment of DZ: Vim+ cells 1 type (red arrows), Vim+ cells 2 type (black arrows); a large aggregation of Vim− cells (black inset); dorsal matrix zone (DMZ) (black oval), granular layer (Grl), other designations see in Figure 2A. (**C**) Magnified fragment of DZ: Vim+ granules are outlined by orange rectangle, Vim+ granules (orange arrows). (**D**) Parenchymal part of the molecular layer of DZ, Vim+ cells 1 type (red arrows); clusters of Vim+ cells 2 type (black arrows) are outlined by red ovals (I and II). (**E**) Granular layer, Vim+ cells 1 type (red arrows); Vim+ cells 2 type (black arrows) and granules localized both inside cells and in the intercellular space (black inset); DMZ is outlined by black oval; ganglionic layer (Gll). (**F**) A focus of additional vimentin expression in the molecular layer of the DZ (in dashed oval). (**G**) Lateral zone: Vim+ cells 1 type (red arrows), Vim+ cells 2 type (black arrows). (**H**) Basal zone (see designations in Figure 2G). (**I**) Granular eminences see designations in Figure 2E. (**J**) Quantitative proportion of Vim+ cells in control and after traumatic injury. (*n* = 5 in each group; * *p* ≤ 0.05 and ** *p* ≤ 0.01 is a significant difference vs. control groups). Student–Newman–Keuls test * chive *p* ≤ oup; * a Newman–Keuls test. IHC labeling of vimentin. The blue rectangle in the pictogram indicates the corresponding area in the micrograph. Scale bars: (**A**,**B**,**D**–**G**) 100 μm; (**C**) 50 μm.

At two days after TBI, we observed significant rearrangements of vimentin immunopositivity in the cerebellum of juvenile *O. masou*. Along with a significant increase in the intensity of immunolabeling of cells and granules, rearrangements of patterns of localization of immunonegative cells occurred in almost all zones. However, the greatest changes were found in the DZ (Figure 2A).

In the DZ after the injury, there was a significant increase in the number of Vim− cells compared to that in the intact animals; in the PVZ and SVZ, the number of immunonegative cells of the neuroepithelial type increased significantly; in the DZ, multiple concentric patterns of localization of these cells were revealed (Figure 2A). In the PVZ, neuropil-like structures and separate intensely labeled Vim+ cells of types 1 and 2 were identified as part of reactive immunonegative formations of a surface and parenchymal localization (Figure 2A, Table S1). Along with the cellular pattern, a granular pattern of Vim+ immunolocalization in the SVZ and PZ was increased (Figure 2A). In the dorsal matrix zone (DMZ) area after the injury, radially directed groups of immunonegative cells with elongated morphology were identified (Figure 2B). These cells formed dense, elongated strands immediately above the DMZ and included single, highly labeled granules (Figure 2B). An increased density of distribution of Vim+ granules was identified in adjacent areas (Figure 2B). Significant structural changes were detected in the apical part of the DZ (Figure 2C), where extensive patterns of immunonegative cells with individual, intensely labeled Vim+ inclusions were found (Figure 2C, inset in the blue dotted oval). Immediately beneath the apical zone, there was an aggregation of type 1 and type 2 Vim+ cells (Figure 2C, Table S1) alternating with extracellular forms of vimentin deposition (Figure 2C). Several foci containing clusters of intensely labeled Vim+ cells were identified in the DZ near the damaged zone (Figure 2D). This area was also characterized by an increased concentration of diffusely located Vim+ cells of a parenchymal localization, combined with clusters of intensely labeled Vim+ granules (Figure 2D).

In the granular layer ventrally adjacent to the DMZ, a moderately labeled Vim+ fiber neuropil was identified after the injury (Figure 2E, black inset). The cells in this region contained intensely labeled Vim+ cytoplasmic inclusions (Figure 2E). At the caudal level, extensive regions of intensely labeled Vim+ granules and cells were identified in the molecular layer, forming extensive diffuse-type layers that proliferated along the molecular layer (Figure 2F). In the lateral (Figure 2G) and basal (Figure 2H) zones, RNNs of heterogeneous composition were revealed, similar in structure to those of the dorsal zone (Figure 2D). However, RNNs in LZ and BZ contained a smaller number of Vim+ cells and were usually characterized by smaller sizes. Thus, the maximum post-traumatic rearrangements of vimentin immunolocalization were detected in the DZ and medial parts of the cerebellar body. In the Gr em region, two types of intensely and moderately labeled Vim+ cells (Figure 2I, inset), as well as a fragmented moderately labeled neuropil spreading among Vim-negative cells, were identified (Figure 2I).

The results of the quantitative analysis showed that after TBI the number of Vim+ cells increased significantly in the DZ, LZ, and in granular eminences (*p* ≤ 0.05) (Figure 2H). A significant increase in Vim+ cells was detected in the BZ of the molecular layer and in the granular layer (*p* ≤ 0.01) (Figure 2J).

### 3.2. Labeling of Neuronal Progenitors with Nestin in the Cerebellum of Intact Juvenile O. masou and at Two Days after TBI

**Figure 3:**
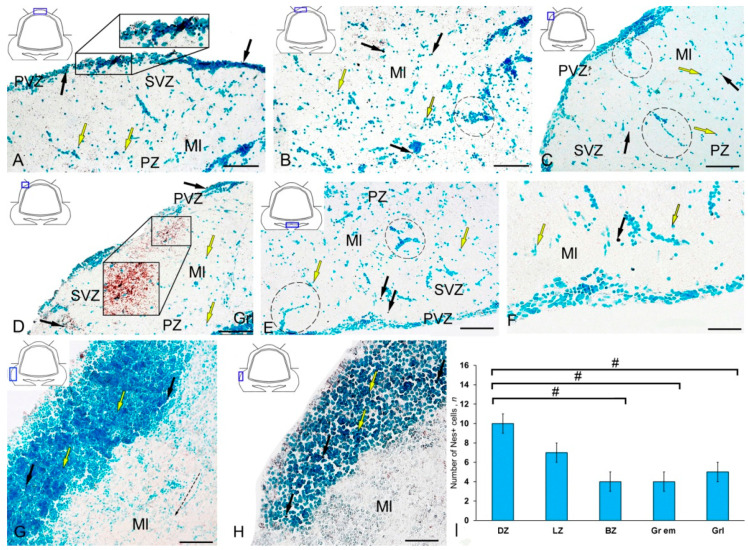
Distribution of nestin-immunopositive cells in the intact cerebellum of juvenile masu salmon *Oncorhynchus masou*. (**A**) General view of the dorsal zone (DZ) of the intact cerebellum: Nes+ cells (black arrows) and Nes− cells (yellow arrows); an aggregation of Nes+ cells in the periventricular zone (PVZ) of the molecular layer (black inset); subventricular zone (SVZ), parenchymal zone (PZ); molecular layer (Ml). (**B**) Parenchymal part of the molecular layer of the DZ: Nes− cell cluster (black dotted oval). (**C**) General view of the lateral zone (LZ) of the intact cerebellum: Nes+ cells (black arrows) and Nes− cells (yellow arrows); an aggregation of Nes+ cells in the PVZ of the molecular layer (black dotted oval). (**D**) Nes+ cells in the LZ of molecular layer, granular layer (Grl), and other designations see in Figure 3A: aggregation of Nes+ cells in the parenchymal part of the molecular layer (black inset). (**E**) General view of the basal zone (BZ). (**F**) An enlarged fragment of Nes+ cells in the BZ. (**G**) General view of granular eminences: black the dashed arrow indicates the direction of migration of Nes− cells. (**H**) Enlarged fragment of Nes+ cells in granular eminences. (**I**) The comparative distribution of Nes+ cells in different zones of cerebellum in intact fish (*n* = 5 in each group; # are significant intergroup differences) ANOVA. IHC labeling of nestin. The blue rectangle in the pictogram indicates the corresponding area in the micrograph. Scale bars: (**A**–**E**,**G**) 100 μm; (**F**,**H**) 50 μm.

The IHC labeling of nestin in the cerebellum of intact juvenile *O. masou* revealed small-sized neural progenitor cells of type 1, round or oval (type 2), located in the area of the SVZ, PVZ, and PZ of the molecular layer and in the granular layer (Figure 3A–E, Table S2). Another type of Nes+ elements was represented by granules of various sizes and shapes, having both an outside neuroepithelial and intracellular localization, diffusely distributed in the molecular layer. The morphometric parameters of Nes+ elements are given in Table S2. In the DZ, most single Nes+ cells or cells forming small clusters were located in the PVZ of the molecular layer (Figure 3A, inset).

Single Nes+ cells were found in the PZ (Figure 3B); their parameters are given in Table S2. Diffuse aggregations of Nes+ granules were found in the PZ and SVZ (Figure 3A, Table S2). Separate constitutive clusters of Nes-negative (Nes−) cells containing immunopositive inclusions were identified in the dorsolateral regions of the molecular layer in the PZ (Figure 3C). In the LZ, the patterns of radial constitutive migration of immunonegative cells containing single Nes+ granules were quite typical (Figure 3C). In this zone, more localized areas of intragranular nestin immunolocalization were encountered (Figure 3C). Nevertheless, in the caudal regions of the LZ, we found separate morphogenetic fields with an increased density of distribution of Nes+ granules and larger Nes+ labeled granules (Figure 3D, inset). The BZ was dominated by single intensely labeled cells (Table S2), as well as by constitutive weakly immunolabeled cell clusters, including single Nes+ subcellular elements (Figure 3E). In the PZ, a granular pattern of nestin distribution in the PVZ was observed (Figure 3E); in most cases, basophilic cells stained with methyl green contained narrow cytoplasmic nestin inclusions (Figure 3F). A few single Nes+ cells were found in the area of granular eminences (Figure 3G, Table S2). In the area of the molecular layer in Gr em, the patterns of migration of Nes− cells, local immunonegative aggregations, and local diffuse regions of granular Nes+ immunopositivity were quite common (Figure 3G). In the region of the granular layer, the concentration of Nes+ cells was low (Figure 3H). The data of quantitative analysis of the distribution of Nes+ cells in the intact cerebellum of juvenile *O. masou* showed the maximum content of Nes+ cells in the DZ, which gradually decreased in the LZ and BZ of the cerebellar body (Figure 3I). The contents of Nes+ cells in the DZ and BZ of the cerebellar body significantly differed (*p* ≤ 0.05). The contents of Nes+ cells in Gr em and in the granular layer also showed significant group differences with that in the DZ (*p* ≤ 0.05) (Figure 3I).

**Figure 4:**
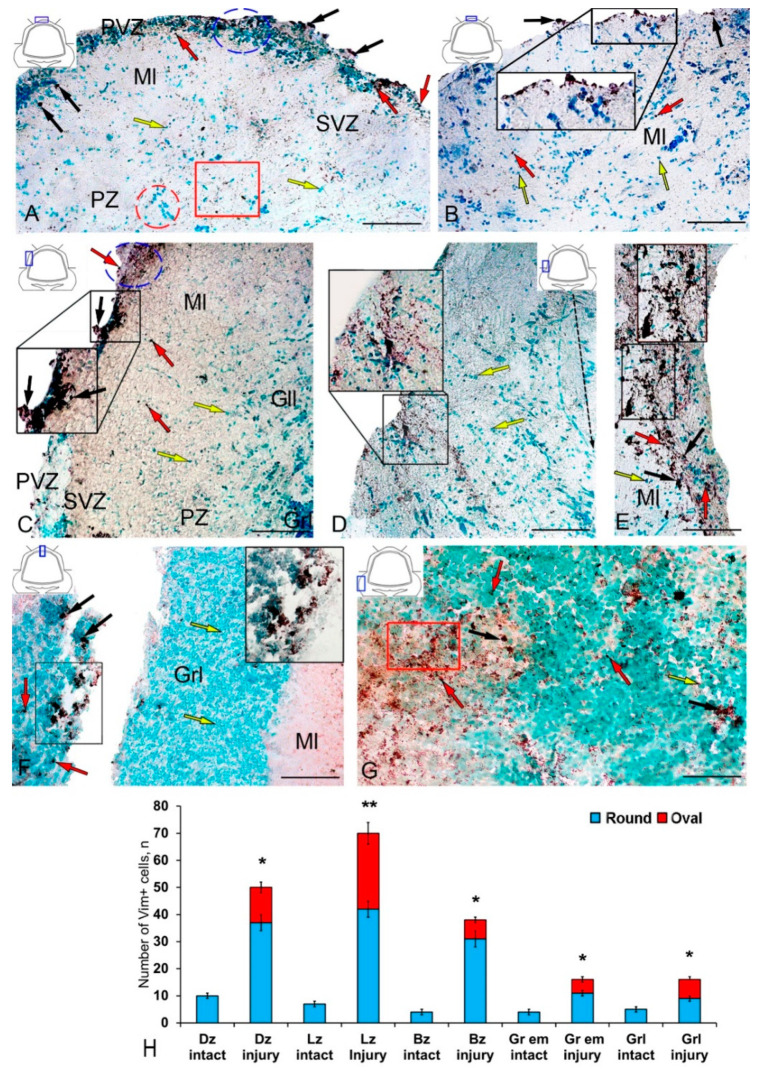
Distribution of nestin in the cerebellum of juvenile masu salmon *Oncorhynchus masou* on day 2 post-injury. (**A**) General view of the dorsal zone (DZ) in the rostral part of the cerebellum: Nes+ cells 1 type (red arrows), Nes+ cells 2 type (black arrows) and Nes− cells (yellow arrows); an aggregation of Nes+ cells (blue inset); an aggregation of Nes− cells (red inset), an aggregation of Nes+ granules (red rectangle); molecular layer (Ml); periventricular zone (PVZ), subventricular zone (SVZ), parenchymal zone (PZ). (**B**) General view of the DZ of the caudal part of the cerebellum: the aggregation of Nes+ cells in the PVZ of the molecular layer (black inset). (**C**) General view of the lateral zone (LZ) in the rostral part of the cerebellum: Nes+ cells in the PVZ of the molecular layer (black inset); an aggregation of Nes+ cells is outlined by the blue dotted oval; ganglionic layer (Gll); granular layer (Grl), and other designations see in Figure 4A. (**D**) LZ of the caudal part of the cerebellum: the black dashed arrow indicates the direction of migration of Nes− cells. (**E**) Large aggregation of Nes+ cells in the LZ (black inset). (**F**) General view of the injury area: asterisk indicates the injury area; a cluster of Nes+ cells in the injury area (black inset. (**G**) Granular eminences: aggregation of Nes+ granules (red rectangle). (**H**) Quantitative proportion of Nes+ cells in control and after traumatic injury. (*n* = 5 in each group; * *p* ≤ 0.05 and ** *p* ≤ 0.01—significant differences vs. control groups). Student–Newman–Keuls test. IHC labeling of nestin. The blue rectangle in the pictogram indicates the corresponding area in the micrograph. Scale bars: (**A**–**G**) 100 μm.

At two days after the TBI to the cerebellum, the nestin immunolocalization patterns differed primarily in the number of Nes+ cells in the PVZ of the molecular layer (Figure 4A,B). Another significant feature was the emergence of a new type 2 of larger Nes+ cells along with constitutive type 1 Nes+ cells (Table S2). In the PVZ of DZ, we found an increase in the total number of neuroepithelial cells forming a hypertrophied layer in the PVZ (Figure 4A). In the acute post-traumatic period, an increase in both Nes+ cells and immunonegative cells was observed in this zone. Separate Nes+ cells were identified in the SVZ and PZ; denser diffuse aggregations of granules were present in the PZ (Figure 4A). In the caudal region of the DZ, the appearance of reactive multidirectional immunonegative aggregations of cells in the state of migration was quite typical (Figure 4B). Streams of migrating cells were combined with heterogeneous Nes+ clusters of granules of different distribution densities.

In the LZ after the injury, we identified rather large, dense (black inset), and loose reactive Nes+ cell clusters (in the blue oval) located in the PVZ (Figure 4C, Table S2). In the SVZ and PZ, the number of single Nes+ cells increased (Figure 4C). In some cases, we identified more complexly organized clusters containing Nes+ and Nes− cells in the PVZ of LZ (Figure 4D). In such clusters, we identified a heterogeneous cellular composition, including immunonegative rounded and elongated migratory cells, as well as intensely labeled Nes+ type 2 cells, in combination with a dense aggregation of Nes+ granules and type 1 cells (Figure 4D, inset). In the same areas, patterns of tangential migration of cells into the PZ and multidirectional cell streams were revealed (Figure 4D). Dense Nes+ clusters of a parenchymal localization in combination with sparse clusters of intensely labeled Nes+ type 2 cells were identified in the LZ in some cases (Figure 4E, inset). In such clusters, the content of immunonegative cells was insignificant, while the PVZ, on the contrary, was dominated by immunonegative cells.

In the area of injury, along the wound canal, we identified clusters of intensely labeled Nes+ type 2 cells and single cells in the granular layer (Figure 4F, inset) located in the immediate vicinity of the injury site (Figure 4F). In the Gr em area, we identified dense diffuse clusters of Nes+ granules in the granular layer and single intensely and moderately labeled Nes+ cells and their clusters (Figure 4G).

The results of the quantitative analysis showed that in the acute post-traumatic period in the cerebellum of juvenile masu, the number of Nes+ cells significantly increases in the DZ and BZ of the cerebellar body, in the granular layer, and in the granular eminences (*p* ≤ 0.05) (Figure 4H). A significant increase in the number of Nes+ cells was found in the LZ (*p* ≤ 0.01) (Figure 4H).

### 3.3. Immunohistochemical Labeling of GS in the Cerebellum of Intact Juvenile O. masou

**Figure 5:**
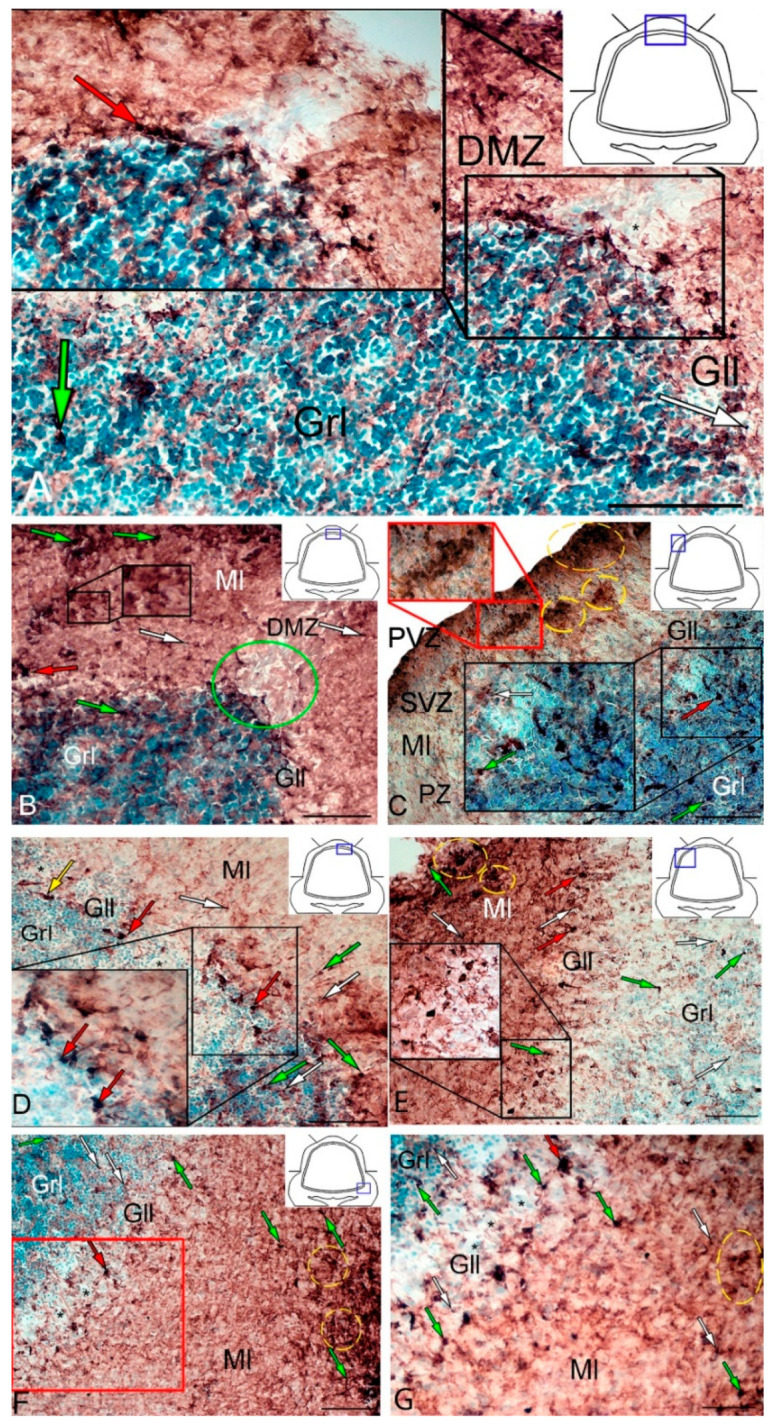
Distribution of glutamine synthetase (GS) in the intact cerebellum of juvenile masu salmon *Oncorhynchus masou*. (**A**) General view of the dorsal zone (DZ) in the rostral part of the cerebellum: GS+ type 1 cells (white arrows); GS+ type 2 cells (green arrows) and GS+ type 3 cells (red arrows); dorsal matrix zone (DMZ) (black inset); ganglionic layer (Gll); granular layer (Grl). (**B**) General view of the DZ of the caudal part of the cerebellum: aggregation of GS+ cells (black inset); DMZ (green oval); molecular layer (Ml), and other designation see in Figure 5A. (**C**) Cluster of type 3 cells in the ganglionic layer of the DZ (indicated by red arrows in the inset). (**D**) Lateral zone (LZ) in the rostral part of the cerebellum: aggregation of GS+ cells in the periventricular zone (PVZ) of the molecular layer (yellow dotted oval); aggregation of GS+ cells in the parenchymal part of the molecular layer (red inset); aggregation of GS+ cells in the granular layer (black inset); GS+ type 1 cells (white arrows); GS+ type 2 cells (green arrows); GS+ type 3 cells (red arrows); the yellow arrow indicates an eurydendroid cell; subventricular zone (SVZ), parenchymal zone (PZ). (**E**) LZ caudal cerebellum: GS+ cells of type 3 in the ganglionic layer (black inset); see designations in Figure 5C. (**F**) Basal zone (BZ): clusters of GS+ cells of the molecular layer (yellow dashed oval); GS− cells are indicated by asterisks. (**G**) Enlarged fragment of BZ in red inset on F. IHC labeling with GS. The blue rectangle in the pictogram indicates the corresponding area in the micrograph. Scale bars: (**A**–**F**) 100 μm; (**G**) 50 μm.

**Figure 6:**
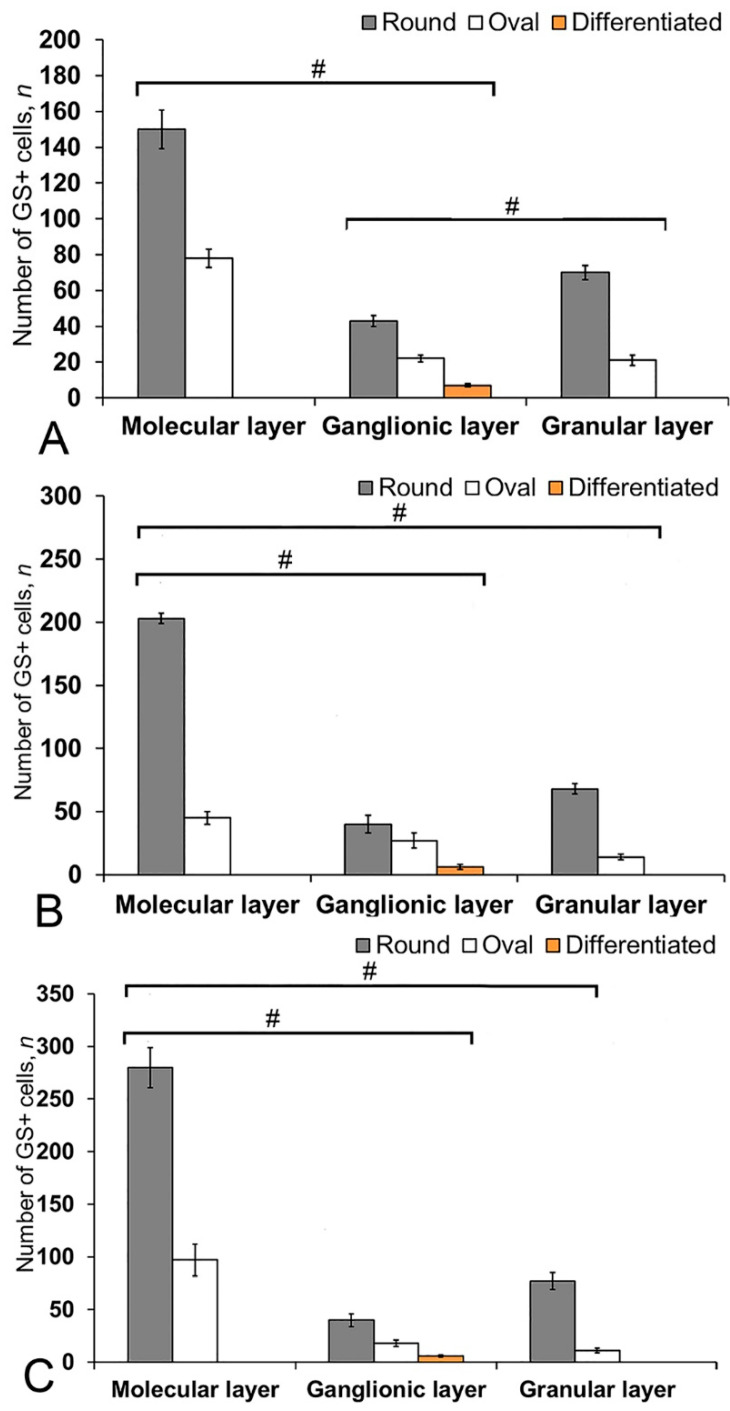
The number of GS+ cells in the intact cerebellum of juvenile masu salmon, *Oncorhynchus masou*. (**A**) Dorsal zone. (**B**) Lateral zone. (**C**) Basal zone. Gray columns are type 1 cells; white, type 2 cells; orange, type 3 cells. #— significant differences (*p* ≤ 0.05) between molecular/ganglionic layers and ganglionic/granular layers (**A**), molecular/ganglionic layers and molecular/granular layers (**B**,**C**, *n* = 5 in each group; # are significant intergroup differences); one-way analysis of variance (ANOVA). The cerebellum layers of juvenile masu salmon, *O. masou*, are plotted on the X-axis.

In the cerebellum of an intact juvenile *O. masou*, the IHC labeling with GS was detected in small, round and oval NE cells, as well as in elongated, larger, type 3 cells (Table S3). In most cases, the intensity of GS immunolabeling in intact animals was moderate or intense (Table S3). In the basal part of the DMZ, an extensive accumulation of GS cells was identified (Figure 5A, inset). In this zone, there were elongated or rounded clusters of type 1 and 2 cells with high labeling intensity (Figure 5B) which extended thin, multidirectional processes into the granular layer. In the areas of the granular layer adjacent to the DMZ, separate intensely labeled GS cells of types 1 and 2 were also detected (Table S3, Figure 5A). The DMZ region itself did not contain GS cells. Moderately/intensely labeled GS cells of type 1 and 2, single, or forming small local clusters, were detected in the molecular layer of the DZ (Figure 5B, inset, Table S3). In some cases, the DZ in the ganglionic layer contained single, intensely labeled GS of type 3 cells with proximal parts GS+ processes (Figure 5A, inset). Piriform cells (Purkinje cells) of the ganglionic layer were GS negative (Figure 5C). The analysis of the quantitative distribution of GS+ cells of various types revealed the largest number of type 1 and type 2 cells in the molecular layer (Figure 6A). The comparative analysis showed intergroup differences between type 1 and type 2 cells of the molecular/ganglionic (*p* ≤ 0.05) and ganglionic/granular layers (*p* ≤ 0.05) (Figure 6A).

In the LZ of the cerebellum, intensely labeled GS clusters of type 1 and 2 cells were identified in the LZ of the molecular layer (Figure 5C, red inset, Table S3). In the granular layer, there were small clusters and single intensely labeled cells, which were larger than most of type 2 cells (Figure 5D, black inset, Table S3). In some cases, GS cells were found in the ganglionic layer, similarly to those in the DZ (Figure 5E, black inset). In the same areas, we identified small clusters of GS cells in the PVZ and SVZ (Figure 5E, yellow oval). The results of the quantitative analysis showed that type 1 cells were the predominant cell type in the molecular layer (Figure 6B). The one-way ANOVA revealed an intergroup difference between the distribution of type 1 and type 2 cells in the molecular/ganglionic (*p* ≤ 0.05) and molecular/granular layers (*p* ≤ 0.05) (Figure 6B). 

In the BZ, the distribution of clusters of type 1 and 2 GS cells was similar to that in the DZ and LZ (Figure 5F). We also detected type 3 GS cells in the ganglionic layer, where the intensity of immunolabeling was, however, slightly lower (Figure 5F, red inset). In some cases, there were aggregations of intensely labeled cells of types 1 and 2 forming local clusters in the granular layer (Figure 5G). The results of the quantitative analysis showed that the number of type 1 cells in the molecular layer was almost three times higher than the number of type 2 cells (Figure 6C). The ANOVA revealed significant differences between the number of type 1 and type 2 cells in the molecular/ganglionic (*p* ≤ 0.05) and molecular/granular layers (*p* ≤ 0.05) (Figure 6C).

**Figure 7:**
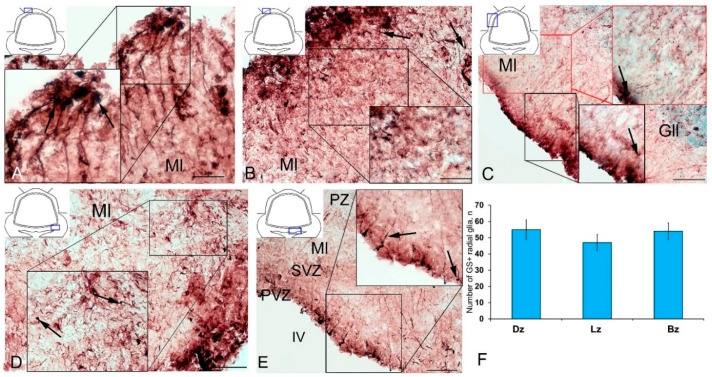
Radial glia (RG) in the intact cerebellum of juvenile masu salmon, *Oncorhynchus masou*. (**A**) DZ of rostral cerebellum: aggregation of GS+ RG cells (black inset); RG cells (black arrows); molecular layer (Ml). (**B**) DZ of caudal cerebellum. (**C**) LZ: GS+ RG cells in the PVZ molecular layer (red arrows); ganglionic layer (Gll). (**D**) BZ in the rostral part of the cerebellum. (**E**) BZ in the caudal part of the cerebellum, periventricular zone (PVZ), subventricular zone (SVZ), parenchymal zone (PZ). (**F**) The number of RGs in the intact cerebellum of juvenile *O. masou*. The cerebellar zones of juvenile *O. masou* are plotted on the X-axis. Dz, dorsal zone; Lz, lateral zone; Bz, basal zone; Ml, molecular layer; Grl, granular layer; IV, fourth ventricle. IHC labeling with GS. The blue rectangle in the pictogram indicates the corresponding area in the micrograph. Scale bars: (**A**–**E**) 100 μm.

Along with GS cells of the NE type, GS cells with radial processes, phenotypically corresponding to RG, were identified in the cerebellum of young intact *O. masou*. Clusters of such cells were detected in the DZ, LZ and BZ (Figure 7A–E). In the DZ, GS+ RG cells were detected in the PVZ of the molecular layer (Figure 7A, black inset). RG processes were observed at a considerable distance in the molecular layer and were either single or organized in bundles (Figure 7A). In some cases, GS cells in the PZ and SVZ formed clusters that lacked visible radial processes; however, in such cases, multidirectional GS fiber fragments were identified in the PZ of the molecular layer (Figure 7B, black inset). In the LZ, the distribution density of such multidirectional fibers was slightly higher than in the DZ (Figure 7C, inset, Figure 7F). In the BZ in the PVZ, the bodies of GS+ RG cells, which extended long, radially oriented processes deep into the parenchyma, were clearly visible (Figure 7D, black inset). In the central part of the BZ, above the roof of the IV ventricle, typical intensely labeled RG cells were identified. Their basal processes extended over considerable distances into the molecular layer, while their apical processes formed terminal end-feet at the ventricular lumen (Figure 7E, inset).

A comparative analysis of the RG distribution showed approximately the same number (55 ± 4) of adult GS+ neuronal stem cells (aNSCs) in the DZ and BZ (Figure 7F); in the LZ, the number of RG cells was slightly lower (Figure 7F). The results obtained are consistent with the morphological data on the localization of the constitutive DMZ and basal matrix zone (BMZ). The ANOVA test did not reveal any significant intergroup differences between the studied areas (Figure 7F).

### 3.4. Immunohistochemical GS Labeling in the Cerebellum of Juvenile O. masou at Two Days after TBI

**Figure 8:**
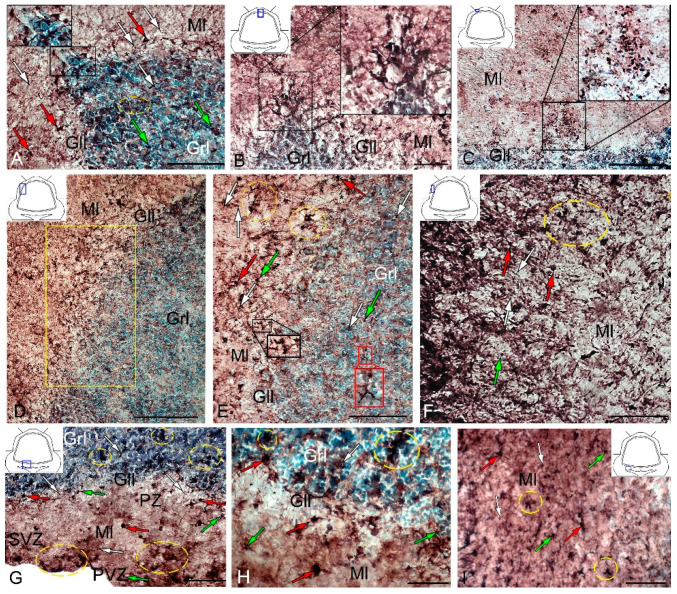
Distribution of GS in the cerebellum of juvenile masu salmon, *Oncorhynchus masou*, on day 2 post-injury. (**A**) DZ: dorsal matrix zone (DMZ) (black inset); GS+ type 1 cells (white arrows); GS+ type 2 cells (green arrows) and GS+ type 3 cells (red arrows); GS– cells are indicated by asterisks; ganglionic layer (Gll); granular layer (Grl); molecular layer (Ml). (**B**) DMZ of the cerebellum, outlined in the black inset. (**C**) Reactive neurogenic niche in the molecular layer of the DZ (black inset). (**D**) LZ in the rostral part of the cerebellum: an enlarged fragment is outlined by a yellow rectangle (**E**). (**E**) The enlarged LZ fragment of the rostral part of the cerebellum, see designations in Figure 8A: aggregation of GS+ cells in the ganglionic layer (black inset); aggregation of GS+ cells in the granular layer (red inset); aggregation of GS+ cells (yellow dashed oval). (**F**) LZ in the caudal part of the cerebellum: yellow dotted line indicates the aggregation of GS+ cells. (**G**) Basal zone (BZ): aggregation of GS+ cells (yellow dotted oval); periventricular zone (PVZ), subventricular zone (SVZ), parenchymal zone (PZ). (**H**) The enlarged fragment of the BZ: cluster of GS+ cells (yellow dashed oval). (**I**) Molecular layer of BZ: cluster of GS+ cells (yellow dashed oval). IHC labeling with GS. The blue rectangle in the pictogram indicates the corresponding area in the micrograph. Scale bars: (**A**–**C**,**E**–**G**,**I**) 100 μm; (**D**) 200 μm; (**H**) 50 μm.

**Figure 9:**
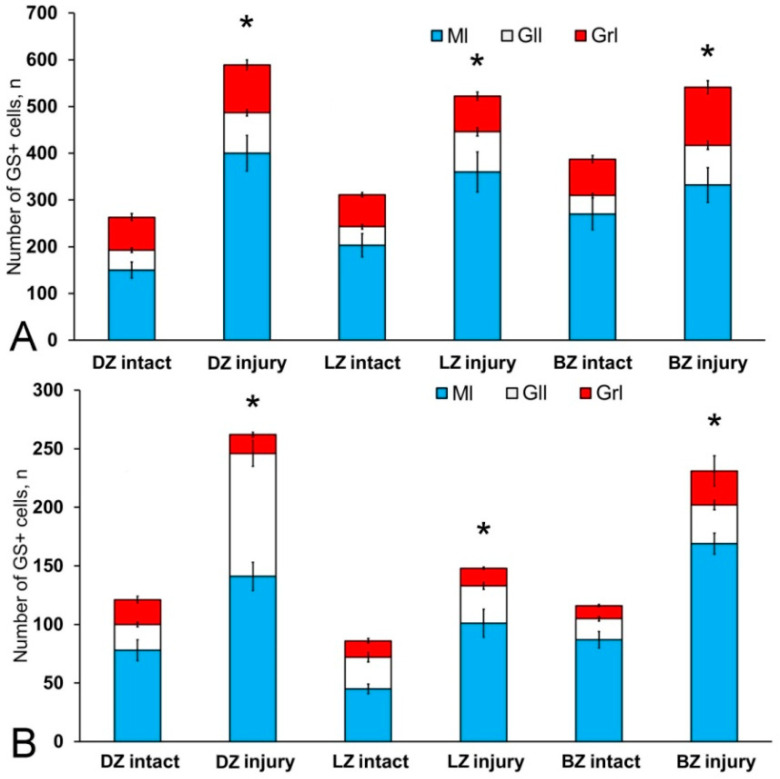
Distribution of GS+ cells in the intact cerebellum of juvenile masu salmon, *Oncorhynchus masou*, and after injury. (**A**) Number of type 1 GS+ cells. (**B**) Number of type 2 GS+ cells. (*n* = 5 in each group; * *p* ≤ 0.05 is a significant difference vs. control groups). Student–Newman–Keuls test. The X-axis indicates the cerebellar area. DZ, dorsal zone; LZ, lateral zone; BZ, basal zone; Ml, molecular layer; Grl, granular layer; Gll, ganglionic layer.

After the TBI, type 1 and type 2 cells were detected in the DMZ; the appearance of type 3 cells with a high intensity of IHC labeling was also recorded (Table S3, Figure 8A, inset). In addition to GS+ cells, short multidirectional GS+ fiber fragments, which formed a medium-density neuropil, were also identified in the DMZ (Figure 8B). Clusters of GS+ cells and fibers extended to the dorsal granular layer, where they alternated with immunonegative granular cells (Figure 8A). In the caudal region, the distribution density of GS+ cells in the DMZ was higher compared to the region located rostrally of the caudal one (Figure 8B, inset). In the area of the ganglionic layer in the DZ, type 3 GS+ cells with intense immunolabeling were revealed (Figure 8A), and Purkinje cells were immunonegative. In the granular layer, along with intensely labeled type 1 cells, larger intensely labeled type 3 cells also appeared (Figure 8A,B, Table S3). In some cases, reactive neurogenic niches (RNNs), containing clusters of intensely labeled GS+ type 1 NE cells, were identified within the molecular layer (Figure 8C, inset). Such clusters had a diffuse structure and differed in size. Quantitative data showed a significant increase (*p* ≤ 0.05) in the number of type 1 cells in the molecular, ganglionic and granular layers in the post-traumatic period (Figure 9A). In the ganglionic layer, the number of type 2 cells increased significantly (*p* ≤ 0.05) (Figure 9B). The LZ had a significantly increased number of type 1 GS+ cells (*p* ≤ 0.05) in the molecular, ganglionic, and granular layers (Figure 8D and Figure 9A, Table S3).

Within the ganglionic layer, there were local dense clusters of type 1 and type 2 cells (Figure 8E, black inset), as well as single or diffusely organized GS+ cells (Figure 8E, yellow oval). GS+ cells formed small clusters and diffusely organized groups in the molecular layer (Figure 8F, in the yellow oval). Single type 1 and type 2 GS+ cells dominated the granular layer. In the molecular layer, the density of distribution of cells in the caudal areas was increased, and local clusters of GS+ cells, as well as numerous single, diffusely organized type 1 cells, dominated (Figure 8F, oval). In the molecular layer, the number of oval type 2 cells increased significantly (*p* ≤ 0.05) (Figure 9B).

In the BZ, after the injury, the number of type 1 GS+ cells significantly increased in the molecular, ganglionic and granular layers (Figure 8G,I and Figure 9A, Table S3). Such cells usually formed RNNs of various volumes and had a both superficial and parenchymal localization (Figure 8G, yellow oval). Larger cells, usually single or paired, appeared in the PZ of the molecular layer, in the ganglionic and granular layers (Figure 8G,H). In the area of the granular layer, the neuropil of moderately labeled GS+ fibers was clearly visible (Figure 8H). In general, the number of oval type 2 cells increased significantly in the BZ in the molecular and ganglionic layers after the injury compared to the control (Figure 8H,G and Figure 9B).

**Figure 10:**
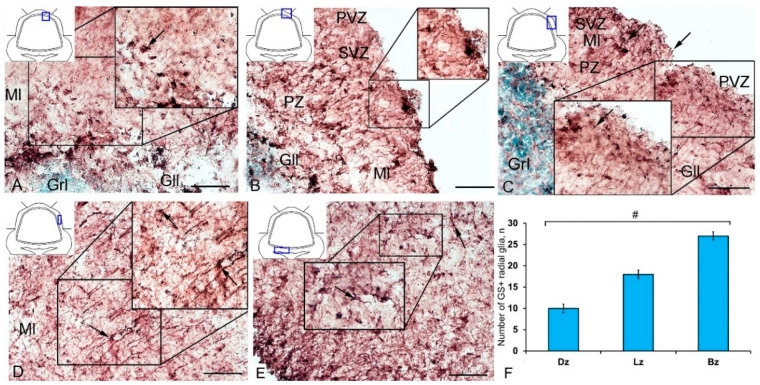
GS+ radial glia (RG) in the injured cerebellum of juvenile masu salmon, *Oncorhynchus masou*. (**A**) DZ of the rostral part of the cerebellum: GS+ cells and RG fibers (black inset); ganglionic layer (Gll); granular layer (Grl); molecular layer (Ml). (**B**) DZ in caudal part of the cerebellum: neurogenic niche containing GS+ cells (black inset); periventricular zone (PVZ), subventricular zone (SVZ), parenchymal zone (PZ). (**C**) LZ in the rostral part of the cerebellum, see designations in Figure 10A. (**D**) LZ in the caudal part of the cerebellum. (**E**) BZ of the cerebellum. (**F**) The number of RG in the damaged cerebellum of juvenile *O. masou*. (*n* = 5 each group) # are ≤ 0.05, significant intergroup differences; one-way analysis of variance (ANOVA). The X-axis indicates the cerebellar areas of juvenile *O. masou*. Dz, dorsal zone; Lz, lateral zone, Bz, basal zone; Ml, molecular layer; Grl, granular layer. IHC labeling with GS. The blue rectangle in the pictogram indicates the corresponding area in the micrograph. Scale bars: (**A**–**E**) 100 μm.

After the TBI, the patterns of GS+ RG labeling changed in the cerebellum of juvenile *O. masou*. In particular, RG processes were visible in the DZ in the area of the ganglionic layer (Figure 10A). In the molecular layer, patterns of RG processes penetrating the PZ of the molecular layer were visible in the RNNs region (Figure 10B, inset) and in the major part of the molecular layer. The total number of RG cells in the DZ significantly decreased in the acute post-traumatic period compared to that in the intact animals. In the LZ, multiple RG fibers were visible in the parenchymal layers of the molecular layer (Figure 10C, inset); a multidirectional moderately labeled neuropil was found in the granular layer (Figure 10C). In some cases, a dense network of RG processes was observed in the PZ of the LZ of the molecular layer, along which we identified single intensely labeled type 1 cells (Figure 10D, inset). As a result of the injury, the number of RG cells decreased in all areas, while the proportion of RG in the lateral zone was significantly higher than in the DMZ and BMZ. Nevertheless, the number of RGs in the BZ significantly (*p* ≤ 0.05) exceeded that in the DZ (Figure 10F). We suggest that the lateral zone retained a greater number of glial-type aNSCs than the DMZ and BMZ, which have a large amount of GS+ radial glia under constitutive conditions.

### 3.5. Immunohistochemical Labeling of CBS in the Cerebellum of Intact Juvenile O. masou

**Figure 11:**
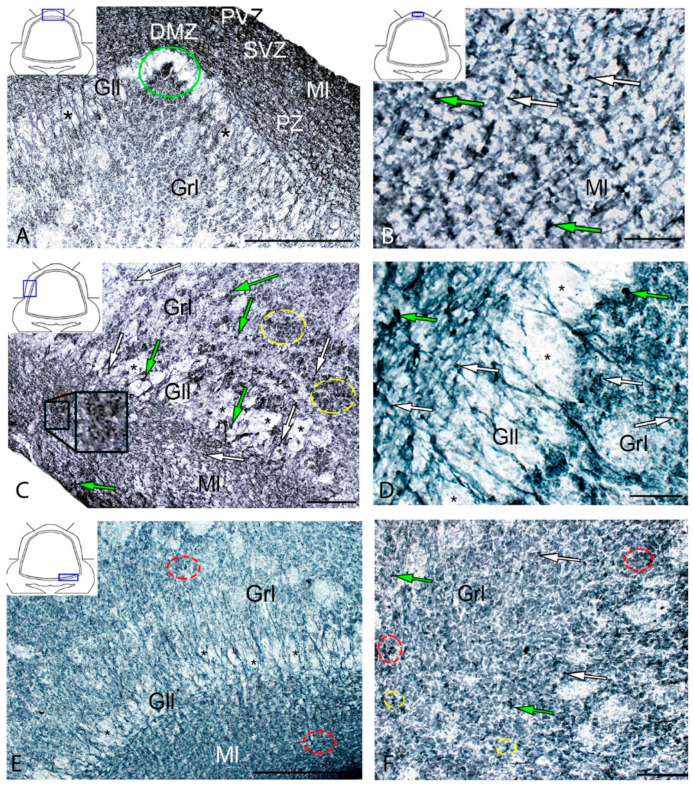
Distribution of cystathionine-β-synthase (CBS) in the intact cerebellum of juvenile masu salmon, *Oncorhynchus masou*. (**A**) General view of DZ: CBS− cells are indicated by asterisks; DZM is outlined by green oval. (**B**) DZ, molecular layer: CBS+ type 1 cells (white arrows); CBS+ type 2 cells (green arrows); periventricular zone (PVZ), subventricular zone (SVZ), parenchymal zone (PZ). (**C**) LZ, see designations in Figure 11A: aggregation of CBS+ cells in the parenchymal part of the molecular layer (black inset); aggregation of CBS+ cells in the granular layer (yellow dashed oval). (**D**) Ganglionic layer: CBS− cells are indicated by asterisks, see designations in Figure 11A. (**E**) Basal zone: CBS− cells are indicated by asterisks; clusters of CBS+ cells (red dashed oval). (**F**) Granular layer; clusters of CBS+ cells (red dotted oval); paired cells (yellow dotted oval). IHC labeling with CBS. The blue rectangle in the pictogram indicates the corresponding area in the micrograph. Scale bars: (**A**) 200 μm; (**B**,**D**) 50 μm; (**C**,**E**,**F**) 100 μm.

In the intact animals, CBS+ cells were detected in the DMZ (Figure 11A), as well as in the molecular and granular layers (Table S4). In the DZ, CBS+ fibers were identified in the molecular and granular layers (Figure 11A).

**Figure 12:**
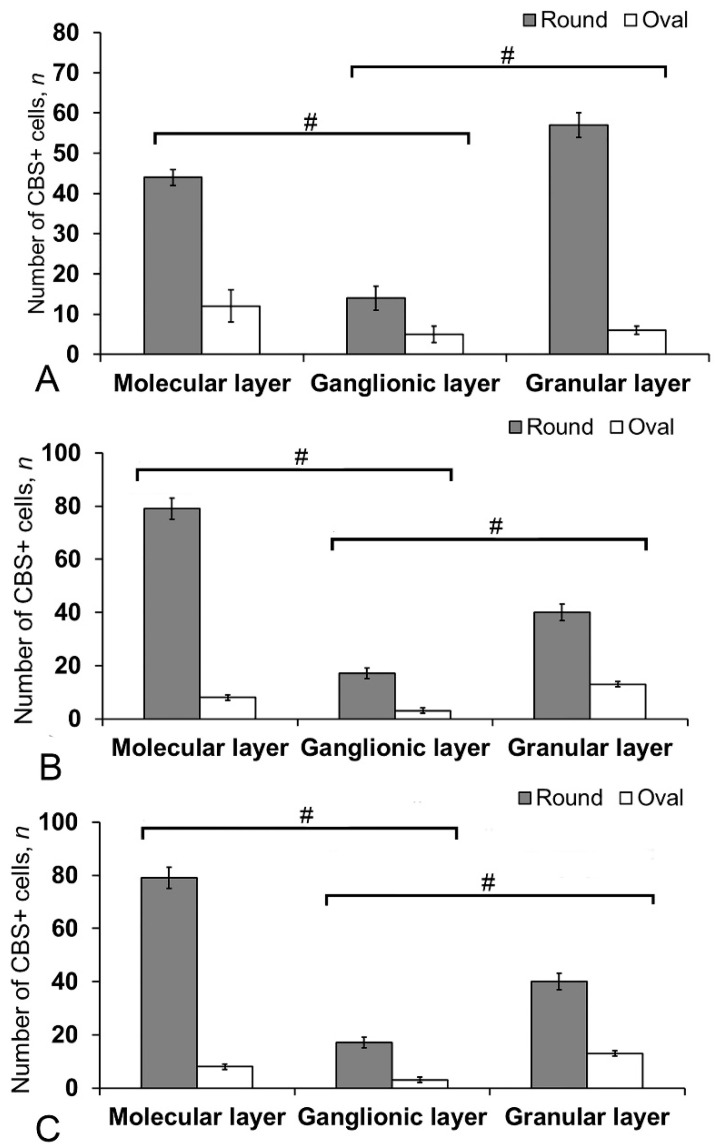
The number of CBS+ cells in the intact cerebellum of juvenile masu salmon, *Oncorhynchus masou*. (**A**) Dorsal zone. (**B**) Lateral zone. (**C**) Basal zone. Gray columns are CBS+ type 1 cells; white, CBS+ type 2 cells. # significant intergroup differences (*p* ≤ 0.05) between molecular/ganglionic layers and ganglionic/granular layers (**A**–**C**), (*n* = 5 in each group; # are significant intergroup differences); one-way analysis of variance (ANOVA). The X-axis indicates the cerebellum layer of juvenile *O. masou*.

Most cells in the molecular layer were grouped into local small clusters or were singular (Figure 11B). The quantitative ratio of type 1 and type 2 CBS+ cells in the molecular, ganglionic, and granular layers is shown in Figure 12. The comparative analysis showed intergroup differences between type 1 and type 2 cells of the molecular/ganglionic (*p* ≤ 0.05) and ganglionic/granular layers (*p* ≤ 0.05) (Figure 12A).

In the LZ, type 1 cells formed diffuse clusters in the molecular layer (Figure 11C, inset) and local clusters in the granular layer (Figure 11C, yellow oval). In the ganglionic layer, type 1 and type 2 cells were also identified (Figure 11D). However, projection neurons (Purkinje cells and eurydendroid cells (EDC)) were CBS-immunonegative (CBS−) (Figure 11D). The quantitative ratio of type 1 and type 2 cells in the LZ is shown in Figure 12B. One-way ANOVA showed intergroup differences in the molecular/ganglionic and ganglionic/granular layers (*p* ≤ 0.05) (Figure 12B).

In the BZ, the patterns of distribution of type 1 and type 2 CBS+ cells were similar to that in other areas (Figure 11E). We identified CBS+ fibers in the BZ crossing the basal part of the molecular, ganglionic, and granular layers (Figure 11E). Diffuse clusters of CBS+ cells (Figure 11F in the red oval), sizes of which were significantly smaller than in the DZ and LZ, were revealed in the molecular and granular layers. In the granular layer, the density of distribution of clusters with type 1 CBS+ cells was significantly lower than in the LZ and DZ (Figure 11F). Type 2 cells were single or paired (Figure 11F, yellow oval). The distribution of type 1 and type 2 CBS+ cells was generally similar to that in the LZ (Figure 12C).

### 3.6. Immunohistochemical CBS Labeling in the Cerebellum of Juvenile O. masou at Two Days after TBI

**Figure 13:**
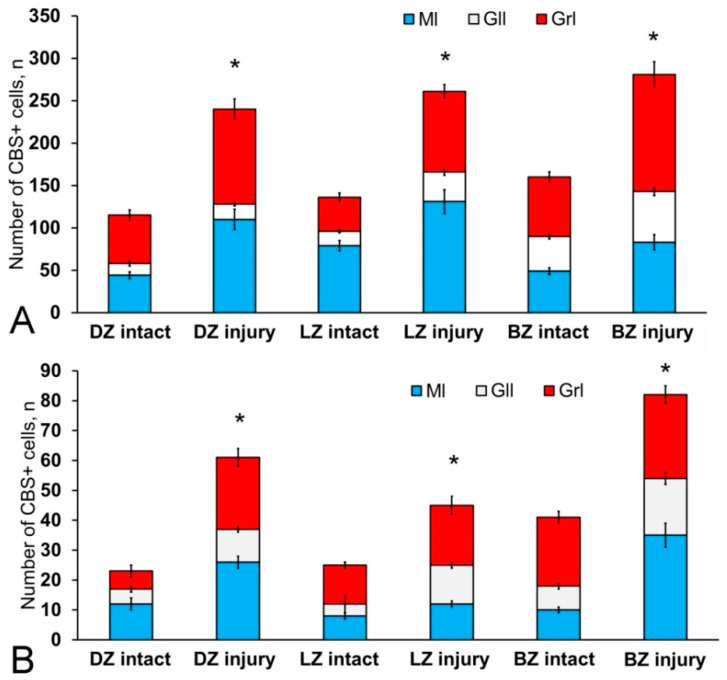
Distribution of CBS+ cells in the intact cerebellum of juvenile *Oncorhynchus masou* and after injury. (**A**) Proportion of round, type 1 CBS+ cells in intact animals and after injury. (**B**) Proportion of oval CBS+ type 2 cells in intact animals and after injury (*n* = 5 in each group; * *p* ≤ 0.05 is a significant difference vs. control groups). Student–Newman–Keuls test. The X-axis indicates the cerebellar area. DZ, dorsal zone; LZ, lateral zone; BZ, basal zone; Ml, molecular layer; Grl, granular layer; Gll, ganglionic layer.

**Figure 14:**
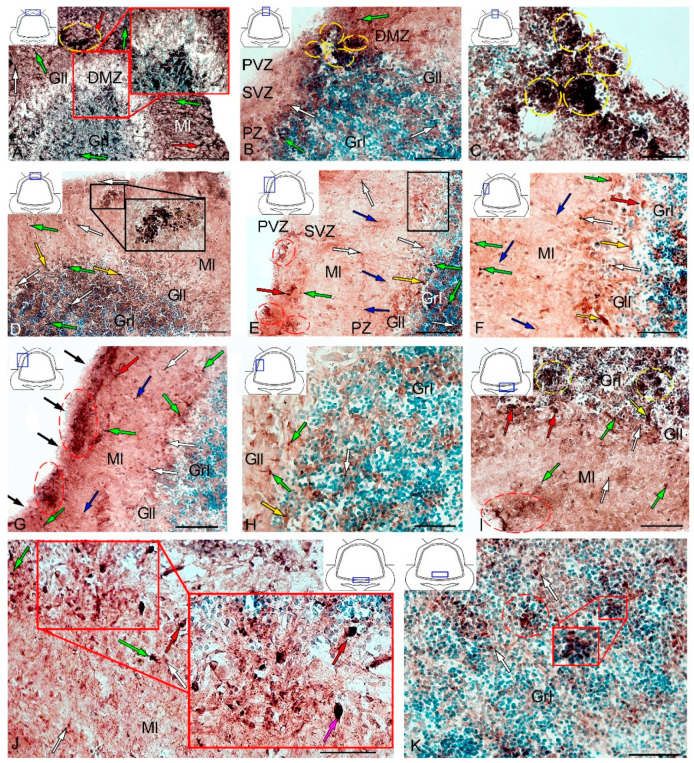
Distribution of CBS in the cerebellum of juvenile masu salmon, *Oncorhynchus masou*, on day 2 post-injury. (**A**) General view of the dorsal zone (DZ) in the rostral part of the cerebellum: dorsal matrix zone (DMZ) (red inset); CBS+ type 1 cells (white arrows); CBS+ type 2 cells (green arrows) and CBS+ 3 cells type (red arrows); aggregation of CBS+ cells (yellow dotted oval); ganglionic layer (Gll); granular layer (Grl); molecular layer (Ml). (**B**) General view of the DZ in the caudal part of the cerebellum: aggregations of CBS+ cells in the DMZ (yellow dashed oval), periventricular zone (PVZ), subventricular zone (SVZ), parenchymal zone (PZ), and other designations see in Figure 14A. (**C**) Area of injury, see designations in Figure 14B. (**D**) DZ containing reactive neurogenic niches with CBS+ cells in their composition (black inset): an aggregation of CBS+ cells in the PVZ of the molecular layer (red dotted oval. (**E**) General view of the LZ in the rostral part of the cerebellum: eurydendroid cells (yellow arrows); migrating CBS− cells (blue arrows); aggregations of CBS+ cells in the PVZ of the molecular layer (red dashed oval); the fragment shown in Figure 14F is outlined by black rectangle. (**F**) The enlarged fragment of the LZ in the rostral part of the cerebellum: eurydendroid cells (yellow arrows); migrating CBS− cells (blue arrows). (**G**) General view of the LZ in the caudal part of the cerebellum: migrating CBS− cells (blue arrows); CBS− cells in the surface layer (black arrows); aggregations of CBS+ cells in the PVZ of the molecular layer (red dashed oval). (**H**) Granular layer of the LZ part of the cerebellum: see designations in Figure 14A. (**I**) BZ: aggregation of CBS+ cells in the PVZ of the molecular layer (red dashed oval); aggregation of CBS+ cells in the granular layer (yellow dotted oval). (**J**) Granular layer of the BZ: aggregation of CBS+ cells (red dotted oval); aggregation of CBS+ cells (red inset); Purkinje cells (pink arrows). (**K**) Granular layer of the BZ (red inset). IHC CBS labeling. The blue rectangle in the pictogram indicates the corresponding area in the micrograph. Scale bars: (**A**) 200 μm; (**B**–**G**,**I**–**K**) 100 μm; (**H**) 50 μm.

After the TBI, the number of CBS+ cells in all areas of the cerebellum increased significantly (Figure 13). In the DMZ, multiple clusters of CBS+ type 1 cells were visible in the apical area of granular layer (Figure 14A, red inset, Table S4). Similar clusters appeared in the molecular layer with CBS+ type 1 cells of a parenchymal localization (Figure 14A, yellow oval). In the PVZ and SVZ of the molecular layer, the number of separate CBS+ type 1 and 2 cells increased (Figure 13 and Figure 14A). In addition, larger type 3 cells appeared, which were absent in intact animals (Figure 14A, Table S4). Large clusters of CBS+ type 1 cells localized in the PVZ and SVZ were identified in the dorsal part of the molecular layer (Figure 14B). In more caudally located regions of the DMZ, local aggregations of CBS+ type 1 cells were found in the molecular and granular layers (Figure 14C, yellow oval). In more distant regions in the molecular layer, single moderately/strongly labeled type 2 cells were identified (Figure 14D). In the same areas, we observed clusters with intensely labeled CBS+ cells of heterogeneous composition, containing cells of types 1 and 2 (inset, Figure 14D). The results of the quantitative analysis showed a significant increase (*p* ≤ 0.05) in the number of CBS+ type 1 cells in the molecular and granular layers of the dorsal zone (Figure 13A).

In the LZ, the number of moderately/intensely labeled CBS+ cells of types 1 and 2 similarly increased (Figure 14E, Table S4). More intensely labeled type 1 cells formed local clusters in the PVZ and SVZ (Figure 14E, red oval); larger moderately labeled type 2 cells were evenly distributed in the PZ of the molecular layer. In the ganglionic layer, there were CBS+ bipolar cells, each having a long process extending deep into the granular layer (Figure 14E, black rectangle). We identified this cell type as EDC. In the LZ, migration patterns of CBS− cells in the molecular layer were revealed (Figure 14F). Diffuse regions containing CBS+ type 1 and type 2 cells were found in the PVZ and SVZ (Figure 14G). In the granular layer, along with single CBS+ type 1 cells, CBS+ neuropil was detected (Figure 14H). In the BZ, the number of type 1 cells increased significantly (*p* ≤ 0.05) in all neuroanatomical zones in the molecular and granular layers of the cerebellum (Figure 13A). These cells formed local clusters in the PVZ and SVZ (Figure 14I, Table S4). The number of type 2 cells also significantly increased in the LZ and BZ (*p* ≤ 0.05) (Figure 13B).

In the ganglionic layer of the BZ after injury, we identified large, intensely labeled CBS+ pear-shaped cells arranged into a multi-row layer (Figure 14J, Table S4). Taking into account morpho-topographic criteria, we attributed this type of cells to differentiated projection neurons (Purkinje cells and EDC cells, correspondently). Along with differentiated cells in the ganglionic layer, intensely labeled type 2 oval cells were revealed (Table S4). Clusters of type 1 cells (Figure 14K, in the red oval) and, in some cases, heterogeneous clusters containing intensely labeled type 1 and 2 cells (Figure 14K, red inset) were found in the granular layer of the BZ. Moderately labeled CBS+ fibers forming a multidirectional neuropil were identified in the granular layer of the BZ (Figure 14K).

## 4. Discussion

### 4.1. Expression of Vimentin and Nestin in Intact Juvenile O. masou and Changes in Plastic Synthesis During TBI of the Cerebellum

Nestin is a protein of intermediate filaments; it is expressed by NSCs and NPCs in vivo and in vitro in the subventricular zone of the lateral ventricle and in the subgranular zone of the *dentate gyrus* of the hippocampus of the adult mammalian brain [3,33,34,35,36,37,38,39,40]. Vimentin and nestin make up the cytoskeleton of NSCs and NPCs [50,51] and are considered as common markers of astrocytic glia in the vertebrate brain [29,50]. Previous studies on various parts of the brain of juvenile chum salmon, *O. keta*, showed that in the telencephalon [15] and the mesencephalic tegmentum [14], Vim+ cells correspond to NE precursors. The results of the study on chum salmon yearlings also showed that aNSCs/NPCs, phenotypically corresponding to RG cells, are generally not characteristic of the telencephalon and mesencephalic tegmentum of juvenile chum salmon. Nevertheless, studies carried out on older grey mullet *Chelon labrosus* individuals showed that the level of vimentin expression in the brain decreases with age [29]. The data of comparative studies on various vertebrates indicate that during the CNS development the expression of vimentin is replaced by GFAP in reptiles [30], birds [31], and mammals [32]. The results of the study on the cerebellum of adult *A. leptorhynchus* showed the presence of a large number of RG cells in the DMZ [27]. Data of a comparative analysis of vimentin/GFAP expression in the cerebellum of adult *A. leptorhynchus* indicated co-expression of both markers in the DMZ and BMZ. However, in an adult animal, the patterns of the vimentin expression in RG fibers were less distinct than the GFAP expression [27]. The results of double labeling on confocal microscopy showed only insignificant areas of co-localization of these markers in the DMZ region [27].

The absence of Vim+ RG in the cerebellum of juvenile *O. masou* differs from the data of immunolabeling in adult *A. leptorhynchus* [27]. However, in general, it corresponds to the distribution of Vim+ NE cells in the telencephalon and in the mesencephalic tegmentum of juvenile chum salmon [14,15], and also agrees with the data of IHC labeling of vimentin for *D. rerio* [6,21,22]. Thus, the data obtained on juvenile masu salmon confirm the opinion that the population of Vim+ neuronal precursors is heterogeneous and probably has a different IHC signature of molecular markers, characterized by age-related and interspecies differences.

Vimentin, as a marker of embryonic cells, is also associated with neuronal differentiation, as has been shown in in vitro studies [52]. Previous studies showed the involvement of vimentin in the growth and differentiation of neurites in cultured neurons [52]. The results of the study on juvenile *O. masou* indicate that the CNNs contain cells of various morphology and size. In particular, the larger and elongated type 2 cells identified in CNNs can correspond to cell forms at the initial stages of neuronal differentiation. Thus, the detection of these cellular forms in the cerebellum of juvenile *O. masou* generally corresponds to the concept of the involvement of vimentin in neurodifferentiation during constitutive neurogenesis. The maximum number of Vim+ cells in the cerebellum of juvenile *O. masou* was found in the DZ.

The detection of the patterns of extracellular expression of vimentin in the molecular layer of the DZ, LZ, and BZ of the cerebellar body, as well as in granular eminences, agrees with the previously obtained results on the mesencephalic tegmentum of juvenile chum salmon *O. keta* [14]. It has been reported that extracellular forms of vimentin expression should be considered as a new neurotrophic factor that enhances axonal growth and restoration of motor functions after TBI of the spinal cord in mice [53]. Vimentin is an intracellular protein involved in the processes of cell adhesion and migration [54,55,56]. Thus, the study of vimentin expression in the cerebellum of juvenile *O. masou* has revealed intense neurogenic constitutive processes. They are manifested as the presence of a large number of neuronal precursors, single and forming CNNs, as well as the extensive extracellular granular patterns of vimentin expression which acts as a neurotrophic and proneurogenic factor and determines a high level of constructive metabolism in the constitutive neurogenesis in *O. masou*.

The results obtained on the cerebellum of juvenile *O. masou* differ from the data on vimentin immunolabeling in the seven-day post-traumatic period in the *O. masou* telencephalon [15]. In the dorsolateral pallium of juvenile *O. masou*, we identified Vim+ RG cells corresponding to glial-type aNSCs [15]. The absence of cells of this type in the juvenile *O. masou* cerebellum in the earlier post-traumatic period may be due to the fact that within two days post-injury, the reactivation programs of the aNSCs in the cerebellum still remain inactivated. As an alternative hypothesis, a scenario can be assumed where the post-traumatic response in the cerebellum of juveniles *O. masou* involves other signaling mechanisms than in the telencephalon. This hypothesis is supported by the presence of a large number of Vim+ NE cells, as well as large zones containing Vim+ granules and forming areas with increased neurotrophic activity, contributing to the activation of neuroregenerative programs in the area of injury. Similar results were obtained at seven days after TBI of the tegmentum in juvenile chum salmon *O. keta* [14]. In particular, after the injury to the tegmentum, several post-traumatic foci of vimentin expression were identified, including aggregations of Vim+ cells surrounded by diffusely organized areas of extracellular expression, whose level markedly increased in the post-traumatic period [14]. In the granular layer of the cerebellum and in the granular eminences in the post-traumatic period, the number of Vim+ cells that formed RNNs of a parenchymal localization increased manifold. We consider the reactivation of CNNs and the formation of RNNs as an additional mechanism that enhances the production of vimentin in juvenile salmon.

The results of the study on the *A. leptorhynchus* cerebellum showed that the aNSCs population in the DMZ expresses vimentin [57]. A co-expression of GFAP, Sox2, and calcium-binding protein (S100b) was also detected in this zone. CNNs in *A. leptorhynchus* contain non-astrocytic cells corresponding to RG cells, which also express glial markers and a small amount of brain lipopolysaccharide binding protein (BLBP). The data obtained on juvenile *O. masou* does not indicate the glial phenotype of Vim+ cells in the DMZ, which is consistent with the data of the study on *D. rerio*, whose cerebellum was not found to have the glial phenotype of Vim+ cells [4,21,22]. Canonical astrocytic or glial markers such as vimentin, GFAP, BLBP were not identified in the DMZ of *D. rerio* [4,21].

Thus, all of the above post-traumatic events, including mass cell migration, enlargement and globalization of the zones of expression of extracellular vimentin, as well as a multiple increase in the number of cells within RNNs in the granular layer and in granular eminences, should be considered as an acute post-traumatic reaction in the cerebellum of juvenile *O. masou*.

### 4.2. Nestin-Positive Progenitor Cells in the Intact and Damaged Cerebellum of Juvenile O. masou

Nestin is an immunohistochemical marker of multipotent neuronal progenitors and RGs [5]. In studies on vertebrates, nestin has been characterized as an intermediate fibrillar type VI protein characteristic of postmitotic neuroblasts [39,51]. Currently, the molecular mechanisms of nestin action and its involvement in the postembryonic and reparative neurogenesis are poorly known. Studies and characterization of the postembryonic and post-traumatic patterns of nestin localization may contribute to understanding the molecular mechanisms underlying the high plasticity of the fish brain. The involvement of nestin in the constitutive neurogenesis processes and the induction of nestin expression, as observed after injury in the regenerative-competent models, may be a key to understanding the processes of neuronal regeneration, because Nes+ cells give rise to both neurons and glial cells during differentiation [51]. In general, the patterns of nestin expression in the intact cerebellum of juvenile *O. masou* were labeled both as single NSCs of the neuroepithelial type and within the CNNs. No nestin expression in RG cells corresponding to aNSCs was detected in the cerebellum of juvenile *O. masou*, which agrees with the data on nestin immunolabeling in the mesencephalic tegmentum of juvenile chum salmon [14]. Previous studies on the mesencephalic tegmentum of juvenile chum salmon showed a constitutive pattern of distribution of Nes+ cells in the tegmentum of intact animals and a complex post-traumatic pattern of nestin immunoexpression as a result of injury [14]. A sufficiently high level of nestin expression in NCPs with a neuroepithelial phenotype was found in the areas of nestin immunopositivity in the mesencephalic tegmentum of chum salmon, both in the periventricular neurogenic zones and in the parenchyma of the brainstem. This indicates a high level of constitutive neurogenesis in the caudal regions of the brain [14].

In studies on mammals, it was shown that nestin labels RG [5,35]. The results of our studies on the cerebellum in juvenile *O. masou*, in contrast to studies on mammals, showed the presence of Nes+ cells not only in the area of matrix zones (DMZ and BMZ), but also outside them, in particular, in the parenchymal layers of the granular layer, where the patterns of nestin expression were present mostly in the form of subcellular granules. The nestin expression was quite widespread in the area of the DZ and LZ as part of parenchymal regions not associated with the matrix zones. Nestin is almost not expressed by mature neurons [34] and is replaced in mature cells by neuron- and glia-specific proteins [33].

Thus, these areas of the cerebellum of juvenile *O. masou* should be considered from the point of view of the neurogenic potential, comparable to that in the DMZ and BMZ. Nevertheless, the results of a quantitative analysis of the juvenile *O. masou* cerebellum showed that a maximum number of Nes+ cells in the DZ, which indicates the priority of this zone in the implementation of the constitutive neurogenic potential. Studies on *D. rerio* showed that the pattern of nestin immunolocalization corresponds to the patterns of immunoexpression of the proliferative nuclear antigen (PCNA), and together, the neurogenic zones of the brain are labeled [51]. Based on this finding, the authors refer to nestin as a primary marker of proliferating multipotent NSCs. The results of studies on the intact cerebellum of juvenile *O. masou* revealed only one type of Nes+ cells that we also consider as neuroepithelial NSCs. However, in another study, nestin, along with GFAP, is reported as a marker of NPCs in the adult *D. rerio* brain [58]. Studies on the telencephalon of transgenic *D. rerio* revealed patterns of nestin expression in NE cells, but not in RG [58]. A study of the patterns of co-expression of nestin with the NSCs Sox2 marker showed the presence of an Nes+/Sox2− phenotype in aNSCs [34]. Similar Nes+ cells were involved in the regeneration of dopaminergic neurons in animals and were the precursors of the population of dopaminergic neurons in the *substantia nigra* of adult mice [34].

After TBI, the intensity of nestin immunostaining significantly increased in the cerebellum of juvenile *O. masou*, and an additional type (type 2) of larger Nes+ cells appeared. The increase in the number of Nes+ cells was most pronounced in the LZ, which should be considered, apparently, as a species-specific feature of the acute post-traumatic response in the cerebellum of juvenile *O. masou*. In the acute post-traumatic period, we observed a significant increase in Nes+ cells in all areas of the juvenile *O. masou* cerebellum, a rearrangement and reorganization of constitutive patterns of nestin immunolocalization, an increase in patterns of granular nestin immunoexpression in the molecular layer, and numerous radial and tangential patterns of post-traumatic cell migration.

The data obtained differ from the results of nestin immunolabeling in the mesencephalic tegmentum of juvenile chum salmon, where, along with local post-traumatic foci of NE cells, Nes+ RG fibers were also detected [14]. In the cerebellum of *O. masou* in the post-traumatic period, we identified Nes+ cells and granules located along the immunonegative radial glial guides. In some cases, sporadic fragments resembling RG were detected. One of the main features of the post-traumatic process is the appearance of an additional type of nestin-expressing cells and a multiple increase in their number in the area of injury. The finding of radial glial guides adjacent to the injury area can be interpreted as the phenomenon of reactive gliosis, as established in studies on the mesencephalic tegmentum of juvenile chum salmon [59]. Patterns of migrating Nes+ NCPs were identified along the RG fibers. The heterogeneous composition of Nes+ RNNs and post-traumatic aggregations indicates the generalization of nestin expression in the acute post-traumatic period. We consider the emergence of larger Nes+ cells as one of the stages of this process. 

The results of studies on *D. rerio* showed the expression of nestin in NE-type cells in the DMZ region [4,22]. According to Kaslin, NE cells are the main source of neurons in the granular layer [4,21,22]. However, studies on *A. leptorhynchus* did not reveal Nes+ cells in the cerebellum [57]. Constitutive progenitors in the DMZ are suggested to be represented by glial-type cells expressing vimentin, GFAP, s100b, and BLBP [57]. Thus, the complex heterogeneous composition of the population of Nes+ cells, identified in the cerebellum of juvenile *O. masou* during the post-traumatic period, is consistent with the results of studies on other fish species. However, we believe that the phenotypic features of Nes+ NSCs can be determined both by a specific period of postembryonic ontogenesis and plastic properties of the fish CNS, and by external epigenetic factors that affect the features of constitutive proliferation and neuronal differentiation of fish brain cells.

### 4.3. Glutamine Synthetase as a Marker of Radial Glia and a Factor of Neuroprotection in the Intact Cerebellum of Juvenile O. masou

In the cerebellum of juvenile *O. masou*, GS labels a heterogeneous population of neuronal progenitors, having mostly an NE phenotype, along with which precursors of the glial phenotype were identified in the DZ, LZ, and BZ. Progenitors of both types are the main sources of neurons in the *O. masou* brain during the constitutive growth, playing an important role in the CNS homeostasis and plasticity during ontogenesis. The ratio of neuronal and glial progenitors varies between different neuroanatomical areas of the cerebellum. Nevertheless, the maximum concentration of NCPs of both types is present in the DZ, while in the BZ the patterns of distribution of precursor NEs and RGs are characterized by pronounced differences. Along with both types of NSCs, type 2 oval cells were found in the primary proliferative regions (DMZ and BMZ), as well as in all the neuroanatomical zones of the cerebellar body and parenchymal regions of the granular layer. They were characterized by a moderate or high intensity of GS labeling and were part of local neural networks providing neurochemical and homeostatic activity in the cerebellum of juvenile *O. masou*. Large, glia-like cells of type 3 were also identified in the ganglionic layer of all neuroanatomical zones of the juvenile *O. masou* cerebellum (Table S3). The quantitative analysis of GS+ cells of different types showed a similar trend in their ratio in all the neuroanatomical zones (Figure 6A–C).

Thus, GS+ cells in the cerebellum of juvenile *O. masou* were mainly located in the SVZ and PZ of the molecular layer and deep in the granular layer. They were represented by a heterogeneous population of NE cells within CNNs and, to a lesser extent, could correspond to the population of glia-like cells (type 3) involved in glutamate metabolism in the area of the ganglionic layer.

Unlike CNNs containing NE cells, GS+ CNNs with RG were mainly localized in the PVZ of the molecular layer of the DZ. Most of the RG precursors were found in the DZ and LZ. The LZ and BZ contained mainly single RG cells. Thus, a heterogeneous population of precursors of the neuroepithelial and glial types, located within the CNNs, was revealed in the intact cerebellum of juvenile *O. masou*. They differed in their topographic features, cellular composition, and, possibly, proliferative behavior. We believe that the presence of a heterogeneous pool of NEs and glial precursors reflects different neurogenic abilities, as well as plastic properties of various neuroanatomical zones of the cerebellum. Our data are consistent with the results of a study on *D. rerio*, whose brain also exhibited unique cellular and molecular profiles of NSCs/NCPs [16]. A study on *D. rerio* showed that the phenotypic heterogeneity of NSCs profiles may be associated with the regulation of tissue homeostasis and regenerative plasticity within various NSCs populations during the postembryonic development of *D. rerio* [22]. As the main structural elements in the CNS, NSCs of the neuroepithelial and glial types are regulated by a variety of external morphogenetic signals with the expression of specific regulatory genes and the activation of certain genetic cascades [60]. Due to various regulatory signals, cell lines of neuroepithelial and glial progenitors organize the constitutive brain growth in the postembryonic period. However, the details of the regulatory effect and the time frame in which the effect of various morphogenetic factors and neurochemical gradients on postembryonic neurogenesis in various fish species is activated are currently almost unstudied. Nevertheless, the heterogeneous neuroepithelial and glial NSCs/NCPs phenotypes in teleost fish and amphibians persists throughout life. They play a special role in the early embryonic CNS development and also have a stimulating effect on the constitutive neurogenesis in adult animals [16].

To date, there is increasingly more information available to confirm that proportions of neuroepithelial and glial precursors in the brain significantly differ in various fish species [16]. It has been found that NE cells have a high potential for NSCs, because they originate from embryonic NSCs in the early stages of CNS ontogenesis and are characterized by high multipotency, giving rise to various types of neurons and a heterogeneous population of glial cells [16]. An essential feature of the organization of glial cells is their polarization along the apical–basal axis and, in some cases, the presence of a cilium on the apical domain of neuroepithelial cell [20].

NE cells are characterized by two types of mitosis: symmetric, increasing the pool of NSCs; and asymmetric, leading to differentiation [61]. Another structural characteristic of NE cells is the interkinetic nuclear migration, due to which the matrix layer containing the NE cells has the appearance of pseudostratified neuroepithelium [62]. It is now known that this type of migration controls the fate of cell progenitors using various signals, in particular, Notch signaling, which keeps the progenitors in the state of rest [63]. The presence of a large number of NE cells in the CNNs of juvenile *O. masou* indicates a heterogeneous and heterochronous constitutive neurogenic activity. Its intensity and other parameters differ between the neuroanatomical zones and represent the final indicators of neurogenic potential. In adult *D. rerio*, the best-known population of NE progenitors is located in the depression of the cerebellum, where they are polarized and express nestin, Sox2, and other NSCs markers that are atypical for radial glia [4]. These NE cells give rise to intermediate precursors that migrate into the granular layer, where they subsequently differentiate into granular cells [21].

In the cerebellum of juvenile *O. masou*, the nestin expression in the DZ is represented not only by numerous CNNs, but also by rather extensive zones containing granular patterns of Nes+ localization. The presence of Nes+ regions indicates a high neurogenic activity and the stem characteristics of this zone, which agrees with the data on *D. rerio* [21]. The presence of a different ratio of precursors of both types (neuroepithelial and glial) in the neuroanatomical areas (DZ, LZ, BZ) of the juvenile *O. masou* cerebellum, in our opinion, reflects the spatiotemporal pattern of ontogenetic evolution and the gradual replacement of embryonic precursors of neuroepithelial cells by adult-type precursors. The existence of such spatiotemporal relationships of various types of progenitors in the mesencephalon of *D. rerio* has been shown [64].

### 4.4. Distribution of GS at Two Days after TBI in the Cerebellum of Juvenile O. masou

After the injury, a significant quantitative predominance of cells was characteristic of the damaged zone, as well as of the DMZ and BMZ, in the juvenile *O. masou* cerebellum. Type 1 and type 2 NE cells involved in post-traumatic neurogenesis were also characterized by an increased intensity in GS labeling (Table S3). A comparative analysis of the distribution of such cells in the DZ, LZ, and BZ showed a significant increase in type 1 cells in the molecular layer of all zones (Figure 9A), as well as a significant increase in type 2 cells in the DZ and BZ (Figure 9B).

**Figure 15:**
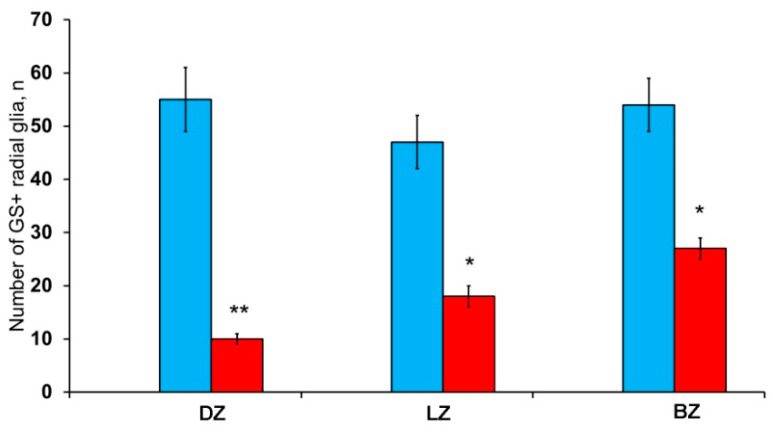
The number of GS+ radial glia (RG) in the intact and injured cerebellum of juvenile masu salmon, *Oncorhynchus masou*. (*n* = 5 in each group; * *p* ≤ 0.05 and ** *p* ≤ 0.01 is a significant difference vs. control groups). Student–Newman–Keuls test. The cerebellum areas of juvenile *O. masou* are plotted on the X-axis. Blue bars are for intact cerebellum, and red bars are for damaged cerebellum. DZ, dorsal zone; LZ, lateral zone; BZ, basal zone.

Cells with the RG phenotype in the acute post-traumatic period were also detected in all the neuroanatomical regions of the cerebellum, however, the results of quantitative analysis showed the maximum RG concentration in the BZ (Figure 10G). Compared with intact animals, the average number of RG cells in all areas of the cerebellum decreased, reaching a minimum level in the DZ (Figure 15).

The post-traumatic localization of RG cells differed from that of intact animals by a lower abundance of RNNs in the PVZ of the molecular layer. RG cell bodies were detected in the SVZ and parenchymal areas of the LZ and BZ without forming clusters. The processes were verified in the thickness of the molecular layer; the most typical patterns of their localization were presented in the LZ. Thus, there is a decrease in the number of RGs in all neuroanatomical zones of the juvenile masu cerebellum in the acute post-traumatic period (Figure 15) when compared with intact animals. The number of NE precursors of types 1 and 2, on the contrary, significantly increases, which indicates the predominance of post-traumatic proliferation of NE precursors compared to RG cells. 

The patterns of radial cell migration along the RG fibers and the formation of numerous RNNs of various volumes and localizations should be considered as another post-traumatic feature. The results of the study on *D. rerio* indicate the presence of a rapidly and slowly proliferating RG population, whose cells divide by asymmetric mitosis [3]. Asymmetric mitoses resulted in the formation of two unequal daughter cells, one of which contains a process [3]. As a result of such asymmetric mitoses in the cerebellum of juvenile *O. masou*, the number of GS+ cells of the NE type could increase. As known, the retraction of the RG cytoplasmic process is observed as a result of TBI in some cases [65], which leads to a decrease in the number of RG cells. RG cells can sometimes retract the process by somal translocation [3], which, in our opinion, could also lead to a decrease in RG in the cerebellum of juvenile *O. masou*. The overall increase in the number of GS+ cells in the cerebellum of juvenile *O. masou* is generally consistent with the concept of post-traumatic gliosis arising after injury in mammals [66,67]. However, the glial scar typical of mammals does not form in the *O. masou* cerebellum. Previous studies have shown an increase in GFAP+ cells and fibers in the cerebellum of juvenile *O. masou* after injury [68]. The results of the experiment with acute post-traumatic injury to the cerebellum in juvenile *O. masou* extend our understanding of the cellular and molecular programs required for successful cerebellar regeneration. 

In the adult *D. rerio*, the processes of regeneration of the brain and spinal cord are provided by the presence of numerous NSCs/NCPs, including various types of precursors such as, in particular, NE, as well as rapidly and slowly proliferating RG [10,69]. Nevertheless, the reparative potential of various types of precursors producing heterogeneous clones of nerve cells, as well as the mechanisms of internal regulation of RNNs in the post-traumatic period of various parts of the brain, still remain unknown. The results of studies on fish have shown the particular value of these models for identifying the mechanisms of functional regeneration of the CNS in vertebrates [7,26,69,70,71]. The presence of a large number of neurogenic niches containing heterogeneous combinations of NSCs/NPCs in the cerebellum of juvenile *O. masou* is a valuable finding that opens up a wide range of opportunities for studying the biology of NE and RG progenitors. Some previous studies provided a simplified view of the biology and further fate aNSCs in the brains of adult teleosts, which assumed that aNSCs retain a high degree of multipotency, which would make it possible to obtain various brain cell lines after damage [9,16,72]. Other studies have suggested the idea of tissue repair exclusively using RG cells. In this case, successful regeneration after TBI could occur with the involvement of RG cells [6,73]. However, taking into account the heterogeneous pattern of the aNSCs population in the forebrain [74,75], in the tectum [16,76,77], as well as the cerebellar and neurogenic hindbrain niches associated with *nervus vagus* [21,78], the question arises as to the cell type which is at the top of the pedigree hierarchy. 

Another question concerns the degree of conservatism in different domains of the aNSCs. Studies on the regenerative potential and biology of forebrain aNSCs [22,79] have shown that certain subtypes of aNSCs have different regenerative abilities and varying molecular control. Whether these properties are a consequence of specific reparative programs or changes in the aNSCs microenvironment as a result of a traumatic process still remains unknown. The heterogeneous pattern of NSCs/NPCs and the different mechanisms of activation of such cells are critically important for the formation of adequate post-traumatic programs associated with regeneration. We believe that an essential feature of the juvenile *O. masou* cerebellum, like various parts of the *D. rerio* brain, is the ability to attract and mobilize various types of NSCs/NPCs. In particular, activation of resting aNSCs of various types is a characteristic sign of ultrastructural and molecular rearrangements, which are observed both under constitutive conditions and after injury [16]. The ability to increase the production of new cells in the damaged fish brain is probably controlled at the aNSCs level or at the NPCs level. The most common strategy for the rapid replacement of lost cells is associated with the elimination of precursors to increase the final amplification pool [16]. The use of enhanced proliferation of progenitors is the most characteristic and distinctive feature for the rapid growth and enlargement of the cerebellum surface in the postembryonic development. In adult rodents, the subventricular neurogenesis in postembryonic development follows a similar scenario [69].

Studies on neurogenic niches in the *D. rerio* cerebellum have shown that RG cells are not the only population of NSCs involved in regeneration. NE cells, in contrast to RG, are the predominant population of progenitors that persist throughout the life of *D. rerio* [21,22]. Fish cerebellum are generally a convenient, relatively simply organized three-dimensional structure, which makes this model suitable for studies on cellular and molecular regenerative features [21,80]. Data on the cerebellum of *D. rerio* indicate the decisive role of the cellular composition of neurogenic niches in the regulation of homeostatic growth and neuronal post-traumatic rearrangements [22]. The growth of the juvenile *O. masou* cerebellum in the first year is more intensive and it contains a greater number of CNNs than in *D. rerio*, therefore we consider this developmental model as more forced, containing a greater number of neurogenic zones with NE precursors. The growth of the cerebellum in the postembryonic period is selective and poorly understood to date, therefore the number of cells mainly increases due to proliferative activity in the granular layer of *D. rerio* [4]. In juvenile *O. masou*, the largest amount of CNNs and RNNs was found in the molecular layer, which indicates significant post-regenerative differences in the NSCs response between juvenile *O. masou* and *D. rerio*. It has been shown that in adult *D. rerio*, only a pool of NE cells is preserved, and PVZ stem cells of RG gradually become immobile or are depleted at juvenile stages [21]. It has been shown by genetic tracing methods that the loss of active RG precursors overlaps with the cessation of creation of some types of neurons, in particular, Purkinje cells [21].

In addition, resting periventricular RG populations correlate with their transformation into epithelium-like cells, which is reminiscent of the transformation of RG into astrocytes in mammals [21]. After a unilateral ablation to the cerebellum, NSCs/NPCs of the NE and RG types are significantly activated [22]. This activation of NE cells leads to a rapid replenishment of the pool of granular cells that were lost as a result of unilateral ablation, while activation of the ventricular RG leads to limited neurogenesis in *D. rerio* [16]. The results of our studies are consistent with the data obtained for *D. rerio* and indicate the predominance of NE type cells as compared to RG in the acute post-traumatic period. 

According to Kaslin’s data, differentiated clones of Purkinje and EDC cells do not regenerate within a month since birth [22]. The specialized Bergman’s glia of the cerebellum, located in the parenchyma, is a static cell form that is poorly involved in the processes of cerebellum regeneration in *D. rerio* [4]. Bergman’s glia demonstrates a very low proliferation rate in juvenile and adult *D. rerio* in the lateral border of the cerebellar CNNs [21]. There is evidence that Bergman’s glia can enter the cell cycle after injury, participating in regeneration, but the sources of the regenerated cells remain unknown. Nevertheless, Bergman’s glial cells largely correspond to the rapidly profiling RG of the tectum [77]. Currently, the molecular signals regulating the constitutive and post-traumatic activity of NSCs in the cerebellum are the least studied as compared to other regions [16,76]. Thus, the results of the conducted studies show the presence of a heterogeneous pool of NE and RG precursors producing GS, which are widespread in the DMZ and BMZ of *corpus cerebelli*, and also have a parenchymal localization. In the acute period of the post-traumatic response, there was a decrease in the number of RG cells and an increase in the number of NE precursors in the neurogenic zones (DMZ and BMZ) and the area of injury, which indicates a specific proneurogenic pattern of the post-traumatic response in the cerebellum of juvenile *O. masou*.

### 4.5. Localization of the H_2_S-Producing System in the Cerebellum of Intact Juvenile O. masou

Currently, the involvement of H_2_S [50], which is one of the three gaseous transmitters along with NO and CO, in ischemic brain damage, control of oxidative stress, and TBI is actively studied [45,81]. H_2_S reactions with many inflammatory mediators, transcription factors, and structural proteins in neurons and glial cells are known [50,82]. However, information on the involvement of H_2_S in neuronal plasticity, adult neurogenesis, and post-traumatic brain injury in regenerative-competent organisms is limited [83]. 

H_2_S is a diffusely spreading gaseous mediator that easily interacts with many signaling molecules, as well as modifies the structure of channel proteins and modulates neuroglia functions [47]. Thus, the high level of H_2_S production in the cerebellum of juvenile *O. masou* correlates with high neurogenic activity in the cerebellum and other parts of the brain, which is consistent with the results previously obtained for trout [12] and other fish species [84]. Studies on the PCNA localization in the cerebellum of *O. masou* showed that the patterns of constitutive proliferation were local, small in size, CNNs located in the area of the DMZ, as well as in the molecular layer of all neuroanatomical zones [68]. The patterns of neurogenesis, revealed using the protein marker of neuronal differentiation HuCD, showed that in intact juveniles the HuCD+ cells are localized within the CNNs of the neuroepithelial type [13]. According to the data obtained on mammals, a high CBS concentration is necessary for the early maturation and growth of neuronal networks, which indicates the involvement of H_2_S in neuronal differentiation [85]. The results of studies on juvenile *O. masou* showed a larger number of cells and a higher level of CBS labeling intensity compared to older trout, *O. mykiss* [12]. This probably indicates more intense neurogenic processes in the cerebellum of juvenile *O. masou*.

The activation of neurons in the cerebellum can lead to the release of glutamate, which, in turn, leads to an increase in intracellular calcium [45,47]. Thus, the wide distribution of type 2 cells in the cerebellum of juvenile *O. masou* is indicative of mediator–modulator intercellular interactions, which is consistent with previously obtained data on fish [12,84]. The high content of type 2 CBS+ cells also suggests intercellular neuron/glial/microglial interactions associated with the release of H_2_S from intensely labeled glia-like cells and/or microglia [86,87].

### 4.6. H_2_S-Producing System in the Cerebellum of Juvenile O. Masou after TBI

As a result of TBI, the patterns of CBS immunolocalization in the cerebellum were significantly altered. Nevertheless, the presence of CBS+ cells in the RNNs of a parenchymal and superficial localization, an increase in the intensity of immunolabeling, as well as a significant increase in type 1 and 2 cells in all neuroanatomical zones of the cerebellum indicate the active involvement of H_2_S in post-traumatic processes. An increase in the volume and number of H_2_S-producing RNNs in the molecular and granular layers unambiguously indicates the involvement of H_2_S in the post-traumatic response associated with neuronal regeneration. Along with neurogenic activity, additional activation of N-methyl-D-aspartate (NMDA) receptors is observed under the conditions of injury. There is an increased release of extracellular glutamate, highly toxic to cerebellar neurons, whose effect is attenuated by the increased production of H_2_S, which acts on voltage-gated ATP-sensitive potassium channels (K_ATP_) and fibrosis transmembrane conductance regulator (CFTR) Cl− channels [88], as well as by the enhanced activation of the glutamate transporter GLT1 [88]. The increase in glutamate excitotoxicity after a cerebellar injury in fish can be neutralized by H_2_S, which reduces the toxic effects of glutamate. The quantitative analysis showed that in all the neuroanatomical zones of the cerebellum, the parameters of CBS+ and GS+ cells are largely consistent (Tables S3 and S4). This suggests a synergistic neutralizing effect of GS (whose production increases manyfold in the post-traumatic period) in type 2 cells and H_2_S, aimed at reducing the toxic effects of glutamate. We consider such effects as neuroprotective, significantly affecting the cellular microenvironment and contributing to the post-traumatic repair.

The results of the study on the cerebellum of juvenile *O. masou* differ from those on the mammalian brain. In the latter, the production of H_2_S in the post-traumatic period after a short-term increase reaches values only to the control level, thereby forming an unfavorable environment for the regenerative processes [81]. It is known that the post-traumatic activation of NMDA receptors in the mammalian brain leads to the activation of cascade caspase-3-dependent pathways leading to apoptosis of mature neurons [89]. The immature cell forms created in the RNNs of the juvenile *O. masou* cerebellum during the post-traumatic period most likely cannot express the full set of NMDA receptors on the cell surface, which prevents the development of the apoptosis response typical of mammals in the cerebellum of juvenile *O. masou*.

We consider the H_2_S-mediated involvement in mitochondrial metabolism during the oxidation of H_2_S-producing enzymes as another post-traumatic effect [90,91]. In their studies, Fu and co-authors demonstrated stress-induced production of H_2_S in mitochondria increasing the production of ATP in cells [92]. Changes in the mitochondrial membrane potential activate the caspase-3-dependent signaling pathway, which can be attenuated by hydrosulfide, which protects neurons from apoptosis [93]. Thus, the neuroprotective and proneurogenic effects of H_2_S in the cerebellum of juvenile *O. masou* are provided by one of the two and/or both pathways leading to an antiapoptotic effect that provides favorable conditions for the neurogenic processes. The results of the study on the cerebellum of juvenile *O. masou* are consistent with the previously obtained results on trout which also revealed the effects of increased H_2_S production in the cerebellum and other parts of the brain after unilateral eye injury [12].

The post-traumatic disturbances in energy metabolism after an injury cause a number of changes in homeostasis resulting from depletion of the intracellular ATP pool. One of the main metabolic changes is glycolysis, which acts as the main reducing factor in the ATP-generating oxidative phosphorylation [94]. The loss of ATP leads to ionic imbalance due to the failure of the activity of ATP-ases or carriers of ATP-dependent ions [81], which regulate the influx of calcium and sodium. The resulting changes cause the potassium outflow due to the subsequent depletion of ATP and calcium accumulation [95,96]. An increase in calcium, in turn, enhances the effects of glutamate and activates calcium-dependent lipases and proteases [96]. Such shifts in ionic homeostasis cause an increase in the production of reactive oxygen species, oxidative stress, and the opening of mitochondrial permeability pores, followed by inflammation and neuronal death [81,97]. In mammals, astrocytes surrounding neurons under normal conditions absorb extracellular glutamate, thus, protecting neurons from excitotoxicity [94,98]. However, in TBI, this may exacerbate ischemic reperfusion injury due to inhibition of the main glutamate transporter GLT1 [99,100].

The results of our studies show that the number of type 3 CBS+ cells in juvenile *O. masou*, on the contrary, significantly increases after a cerebellar injury. In our opinion, they actively metabolize extracellular glutamate, decreasing the intracellular production of reactive oxygen species, thus, eliminating the development of oxidative stress. H_2_S is involved in many signaling pathways that attenuate the effects of oxidative stress, in particular, in the glutathione cycle, activation of enzymes and transcription factors related to the redox balance [85]. Consequently, one of the post-traumatic effects in the cerebellum of juvenile *O. masou* is an increase in H_2_S production, which contributes to the maintenance of the cerebrovascular homeostasis, including antiapoptotic, anti-inflammatory, antioxidant, and proneurogenic effects, and, thus, facilitates reduction in the excitotoxic damage to neurons that occurs during oxidative stress.

## 5. Conclusions

The results of immunolabeling of vimentin in the cerebellum of juvenile *O. masou* revealed the expression of vimentin in small, rounded type 1 cells and larger, oval type 2 cells in all the neuroanatomical regions of the cerebellar body: DZ, LZ, and BZ. Small, rounded type 1 cells, corresponding to NE cells, were also identified in the area of Gr em. In the cerebellum of *O. masou* yearlings, no Vim+ cells with the RG phenotype corresponding to the aNSCs of other vertebrates were found. Thus, most of the heterogeneous Vim+ clusters corresponding to constitutive neurogenic niches (CNNs) were located in the PVZ of the molecular layer in all regions of the cerebellar body. Single cells of parenchymal localization were found both in the molecular layer of the cerebellar body and in the area of granular eminences. Type 2 cells, characterized by larger sizes and elongated morphology, were part of heterogeneous CNNs and were cells at the initial stage of differentiation during constitutive neurogenesis. The third type of Vim+ elements in the cerebellum was represented by Vim+ granules of a subcellular size and extracellular localization. Such elements, as a rule, were grouped into morphogenetic zones of various lengths and were most frequently found in the subventricular and parenchymal regions of the DZ, LZ, and BZ.

Two days post-injury after the TBI of the cerebellum, we observed a significant increase in the number of Vim+ cells and granules. In all the neuroanatomical zones of the cerebellum and in granular eminences, the intensity of vimentin immunolabeling increased. The most significant increase in the number of Vim+ cells was recorded from the BZ of the molecular layer and from the granular layer. The mass migration of cells in the molecular layer and the formation of RNNs and/or reactivation of CNNs, leading to a significant increase in Vim-immunopositivity in the cerebellum, should be considered equally significant events in the acute post-traumatic period. With a multifold increase in the number of Vim+ cells of types 1 and 2, nevertheless, no Vim+ RG patterns corresponding to aNSCs were observed. 

In our studies on the cerebellum of juvenile *O. masou*, patterns of nestin immunopositivity were revealed in all the neuroanatomical zones of the cerebellar body and in granular eminences in rounded small cells of type 1, as well as in noncellular forms of nestin expression, represented by extracellular granules. In the DZ, the most typical patterns were the localization of Nes+ zones, forming superficial and parenchymal CNNs. Areas of extracellular expression of nestin were represented to a lesser extent than patterns of the extracellular expression of vimentin. Nevertheless, such diffusely organized Nes+ clusters formed regions of the molecular layer that were most frequently present in the dorsolateral region and in the zone of granular eminences. In general, the patterns of nestin expression in the intact cerebellum of juvenile *O. masou* were labeled both as single NSCs of the neuroepithelial type and within the CNNs. No nestin expression in RG cells corresponding to aNSCs was detected in the cerebellum of juvenile *O. masou*. 

After TBI, the intensity of nestin immunostaining significantly increased in the cerebellum of juvenile *O. masou*, and an additional type 2 of larger Nes+ cells appeared. The increase in the number of Nes+ cells was most pronounced in the LZ, which should be considered, apparently, as a species-specific feature of the acute post-traumatic response in the cerebellum of juvenile *O. masou*. In the acute post-traumatic period, we observed a significant increase in Nes+ cells in all areas of the juvenile *O. masou* cerebellum, a rearrangement and reorganization of constitutive patterns of nestin immunolocalization, an increase in patterns of granular nestin immunoexpression in the molecular layer, and numerous radial and tangential patterns of post-traumatic NE cell migration. In the area of injury, along the wound canal, numerous local aggregations of Nes+ cells forming RNNs were found. A significant increase in IHC activity was revealed in granular eminences where the patterns of diffuse extracellular nestin expression, which was absent in intact animals, significantly prevailed. The presence of local reactive clusters of Nes+ cells, both in the area of injury and in more distant areas, indicates the proliferation of NSCs with the formation of Nes+ cell groups having a high proliferative/regenerative potential. Such clusters, as a rule, were formed by types 1 and 2 cells. This allows us to consider them as descendants of NCPs which are activated by the traumatic process and are not found in the cerebella of intact animals. We suggest that another feature of the post-traumatic response is the emergence of a large number of intensely labeled cell groups combined with granular patterns. The presence of nestin is inextricably linked with the reorganization of the cytoskeleton and, most likely, reflects to some extent the proliferative activity within the reactive conglomerates. The appearance of such dynamic units along with dense reactive Nes+ clusters of cells indicates an active post-traumatic reorganization of neurogenic zones in the area of injury. 

In the cerebellum of juvenile *O. masou*, GS labels a heterogeneous population of neuronal progenitors, having mostly an NE phenotype, along with which precursors of the glial phenotype were identified in the DZ, LZ, and BZ. Progenitors of both types are the main sources of neurons in the *O. masou* brain during the constitutive growth, playing an important role in the CNS homeostasis and plasticity during ontogenesis. Precursors with an RG phenotype were found in the ventricular region of the DZ, LZ, and BZ of the molecular layer as part of heterogeneous constitutive neurogenic niches. The detection of precursors of both types is an important finding, as it shows a certain relationship in the cerebellum of *O. masou* underyearlings between the embryonic type NSCs present and the aNSCs, which indicates the neurogenic activity of adult animals. 

Nevertheless, the ratio of type 1 and type 2 cells in CNNs in all neuroanatomical zones of the cerebellum in the molecular and granular layers showed a significant predominance of type 1 cells. At the same time, the number of type 2 cells in the composition of heterogeneous CNNs differed, and was the maximum in the BZ and the minimum in the LZ. In the granular layer, similar patterns were revealed, where the number of type 1 cells in the CNNs was 2–2.5 times higher than the number of type 2 cells. Cells of type 3 in all neuroanatomical areas were represented by a more limited population compared to cells of types 1 and 2. Thus, GS+ cells in the cerebellum of juvenile *O. masou* were mainly located in the SVZ and PZ of the molecular layer and deep in the granular layer. They were represented by a heterogeneous population of NE cells within CNNs and, to a lesser extent, could correspond to the population of glia-like cells (type 3) involved in glutamate metabolism in the area of the ganglionic layer.

Unlike CNNs containing NE cells, GS+ CNNs with RG were mainly localized in the PVZ of the molecular layer of the DZ. Most of the RG precursors were found in the DZ and LZ. The LZ and BZ contained mainly single RG cells. Thus, a heterogeneous population of precursors of the neuroepithelial and glial types, located within the CNNs, was revealed in the intact cerebellum of juvenile *O. masou*. They differed in their topographic features, cellular composition, and, possibly, proliferative behavior. We believe that the presence of a heterogeneous pool of NEs and glial precursors reflects different neurogenic abilities, as well as plastic properties of various neuroanatomical zones of the cerebellum.

At two days after TBI of the *O. masou* cerebellum, a significant increase in the intensity of GS labeling of type 1 and type 2 cells was revealed in all the neuroanatomical zones. Furthermore, the induction of activity in cells with elongated morphology (type 3), which are absent in intact animals, was also observed (Table S3). We associate the appearance of such cells with the intensification of glutamate metabolism. Accordingly, type 3 cells are considered by us as glia-like cells, probably involved in the reuptake of glutamate from the intercellular space, whose number increases manyfold after TBI. The largest number of such cells was identified in the DZ adjacent to the injury site. Thus, in order to utilize the toxic effects of glutamate and prevent the increasing excitotoxicity accompanied by an intense neuroimmune reaction of secondary inflammation, type 3 cells were activated in the cerebellum of juvenile *O. masou*, which regulated the cerebellar post-traumatic homeostasis of glutamate. Thus, in the acute post-traumatic period, reorganization of neuroepithelial CNNs and the formation of RNNs heterogeneous in their cellular composition occurred in all areas of the cerebellum. The heterogeneous pattern of NSCs/NPCs and the different mechanisms of activation of such cells are critically important for the formation of adequate post-traumatic programs associated with regeneration. 

Our studies have shown that during the postembryonic period of the cerebellum development in juvenile *O. masou*, type 1 and type 2 cells are CBS-labeled with a high or moderate intensity (Table S4). A high intensity of CBS immunolabeling was detected in the DMZ, as well as in the molecular and granular layer of all neuroanatomical zones of the cerebellum, where small cells of type 1 dominated compared to larger type 2 cells. Such cells most frequently formed local constitutive domains. An analysis of the morpho-topographic properties of these domains showed a high degree of similarity with CNNs containing Nes+ and Vim+ cells. A high degree of similarity between H_2_S-producing aggregates in the cerebellum of juvenile *O. masou* was also revealed with GS-producing CNNs located in the dorsal, lateral, and basal zones. Thus, it is usually assumed that the H_2_S-producing population of type 1 and type 2 cells is involved in the formation of the intercellular environment and modulates the activity of CNNs containing NSCs/NCPs markers. The results of CBS immunolabeling in the cerebellum of an intact *O. masou* suggest that H_2_S synthesized in these regions is diffusely distributed within the molecular and granular layers, influencing the processes of proliferation and neuronal differentiation. In the cerebellar parenchyma, H_2_S-producing cells were identified. They produce H_2_S into the intercellular space, where the differentiation of young neurons and the proliferation of NSCs/NCPs occur, thus, creating the special microenvironment that supports the proliferation of NE and RG precursors, as well as promoting neuronal differentiation. Thus, H_2_S produced in the neurogenic and parenchymal regions of the cerebellum of juvenile *O. masou* can be considered as a proneurogenic factor involved in the constitutive neurogenesis. 

As a result of TBI, the patterns of CBS immunolocalization in the cerebellum were significantly altered. Nevertheless, the presence of CBS+ cells in the RNNs of a parenchymal and superficial localization, an increase in the intensity of immunolabeling, as well as a significant increase in type 1 and 2 cells in all neuroanatomical zones of the cerebellum indicate the active involvement of H_2_S in post-traumatic processes. An increase in the volume and number of H_2_S-producing RNNs in the molecular and granular layers unambiguously indicates the involvement of H_2_S in the post-traumatic response associated with neuronal regeneration. Along with neurogenic activity, additional activation of NMDA receptors is observed under the conditions of injury. There is an increased release of extracellular glutamate, highly toxic to cerebellar neurons, whose effect is attenuated by the increased production of H_2_S, which acts on voltage-gated K_ATP_ and CFTR Cl− channels. The increase in glutamate excitotoxicity after a cerebellar injury in fish can be neutralized by H_2_S, which reduces the toxic effects of glutamate. The quantitative analysis showed that in all the neuroanatomical zones of the cerebellum, the parameters of CBS+ and GS+ cells are largely consistent (Tables S3 and S4). This suggests a synergistic neutralizing effect of GS (whose production increases multifold in the post-traumatic period) in type 2 cells and H_2_S, aimed at reducing the toxic effects of glutamate. We consider such effects as neuroprotective, significantly affecting the cellular microenvironment and contributing to the post-traumatic repair. The immature cell forms created in the RNNs of the juvenile *O. masou* cerebellum during the post-traumatic period most likely cannot express the full set of NMDA receptors on the cell surface, which prevents the development of the apoptosis response typical of mammals in the cerebellum of juvenile *O. masou*. The obtained results allow us to consider H_2_S as a biologically active substance, the numerous known effects of which can be supplemented by participation in the processes of constitutive neurogenesis and neuronal regeneration.

## Supplementary Materials

Supplementary materials can be found at https://www.mdpi.com/1422-0067/21/24/9638/s1.

## Abbreviations

aNSC	adult neuronal stem cells:
ATP	adenosine triphosphate;
BLBP	brain lipopolysaccharide binding protein;
BMZ	basal matrix zone;
BZ	basal zone;
CBS	cystation β-synthase;
CFTR	fibrosis transmembrane conductance regulator (CFTR) Cl− channels;
CNN	constitutive neurogenic niche;
CNS	central nervous system;
DMZ	dorsal matrix zone;
DZ	dorsal zone;
EDC	eurydendroid cells.
GFAP	glial fibrillar acidic protein;
Gll	ganglionic layer;
GLT1	glutamate transporter;
Gr em	granular eminences;
Grl	granular layer;
GS	glutamine synthetase;
H_2_S	hydrogen sulfide;
HuCD	protein of neuronal differentiation;
IHC	immunohistochemical labeling;
K_ATP_	ATP-sensitive potassium channels;
LZ	lateral zone;
Ml	molecular layer;
NE	neuroepithelial
Nes	nestin;
NMDA	*N*-methyl-D-aspartate receptor;
NPC	neuronal progenitor cells;
NSC	neuronal stem cells;
OD	optical density;
PCNA	proliferative nuclear antigen;
PVZ	periventricular zone;
PZ	parenchymal zone;
RG	radial glia;
RNN	reactive neurogenic niche;
S100b	calcium-binding protein;
SVZ	subventricular zone;
TBI	traumatic brain injury;
UOD	units of optical density;
Vim	vimentin;
