# Supplementary material for: Hydrogen Sulfide Modulates Adult and Reparative Neurogenesis in the Cerebellum of Juvenile Masu Salmon, Oncorhynchus masou
Source: Int J Mol Sci. 2020 Dec 17;21(24):9638. doi: 10.3390/ijms21249638 (PMC7766854; doi:10.3390/ijms21249638)
Supplement: Supplementary file 1 [file ijms-21-09638-s001.pdf]

Table S1. Morphometric and densitometric characteristics of Vim-labeled cells (M ± SD) in the intact and injured cerebellum of juvenile masu salmon, *Oncorhynchus masou*.

Brain Areas	Intact Animals			Injured Cerebellum	
	Vimentin				
	Type of cells/Granules	Cell Size, µm	Optical Density, UOD	Cell Size, µm	Optical Density, UOD
**Dorsal zone**	1 type, round<br>2 type, oval<br>Granules	3.5 ± 0.3 / 3.1 ± 0.2 (PVZ, SVZ, PZ)<br>6.1 ± 0.5 /5.2 ± 0.9 (PVZ, SVZ, PZ)<br>2.1 ± 0.1/ 1.9 ± 0.2 (PVZ, PZ)	++\+++<br>+++<br>++	3.6 ± 0.2 / 3 ± 0.2 (PVZ, SVZ, PZ)<br>6.2 ± 0.2 /5.9 ± 0.2 (PVZ, SVZ, PZ)<br>2 ± 0.1/ 2 ± 0.1 (PVZ, SVZ, PZ)	+++<br>+++<br>++
**Lateral zone**	1 type, round<br>2 type, oval<br>Granules	4 ± 0.2/3.6 ± 0.3 (PVZ, SVZ)<br>6.1± 0.5 /5.7 ± 0.6 (PVZ, SVZ)<br>2 ± 0.3/ 2 ± 0.2 (PVZ, SVZ, PZ)	++\+++<br>+++<br>+++\++	3.6 ± 0.4 / 3.9± 0.2 (PVZ, SVZ, PZ)<br>6.5 ± 0.3/ 4.2 ± 0.4 (PVZ, SVZ, PZ)<br>2 ± 0.1/ 1.9 ± 0.1 (PVZ, SVZ, PZ)	+++<br>+++<br>++
**Basal zone**	1 type, round<br>2 type, oval<br>Granules	3.8 ± 0.2 / 3.2 ± 0.4 (PVZ)<br>5.9 ± 0.3/ 5.9 ± 0.2 (PVZ, SVZ)<br>2.2 ± 0.2/ 1.8 ± 0.3 (PVZ, SVZ, PZ)	++\+++<br>+++<br>+	3.4 ± 0.2 / 3.8 ± 0.2 (PVZ, SVZ, PZ)<br>6.1 ± 0.4/ 5.8 ± 0.2 (PVZ, SVZ, PZ)<br>2 ± 0.2/ 2 ± 0.1 (PVZ, SVZ, PZ)	++/+++<br>+++<br>++
**Granular Eminences**	1 type, round<br>2 type, oval<br>Granules	4.3 ± 0.2 / 4.3 ± 0.4<br>–<br>2 ± 0.2/ 1.9 ± 0.1	++\+++<br>–<br>+	4.4 ± 0.2 / 4 ± 0.3<br>5.9 ± 0.2/ 5.6 ± 0.3<br>2.2 ± 0.2/ 1.8 ± 0.2	+++<br>+++<br>++

Optical density (OD) in cells was classified according to the following scale: high (190–150 UOD, which corresponds to +++) and moderate (150–100 UOD, which corresponds to ++). The large and small diameters of the cell body are shown with a slash (/). Cells moderately and intensely labeled are shown with a slash (\). PVZ - periventricular zone, SVZ - subventricular zone, PZ - parenchymal zone.

Table S2. Morphometric and densitometric characteristics of Nes-labeled cells (M ± SD) in the intact and injured cerebellum of the juvenile masu salmon, *Oncorhynchus masou*.

Brain Areas	Intact Animals			Injured Cerebellum	
	Nestin				
	Type of cells/Granules	Cell Size, µm	Optical Density, UOD	Cell Size, µm	Optical Density, UOD
**Dorsal zone**	1 type, round<br>2 type, oval<br>Granules	4.3 ± 0.4/3.7 ± 0.5 (PVZ, SVZ, PZ)<br>–<br>2.3 ± 0.3/1.6 ± 0.4 (PVZ, SVZ, PZ)	+++<br>–<br>+++	4.2 ± 0.3/3.5 ± 0.5 (PVZ, SVZ, PZ)<br>6.7 ± 0.2/5.1 ± 0.5 (PVZ, SVZ)<br>2 ± 0.3/2 ± 0.3 (PVZ, SVZ, PZ)	+++<br>+++<br>+++
**Lateral zone**	1 type, round<br>2 type, oval<br>Granules	3.7 ± 0.3/2.9 ± 0.4 (PVZ, SVZ)<br>–<br>2.1 ± 0.1/1.7 ± 0.3 (PVZ, SVZ, PZ)	+++<br>–<br>++\+++	4.4 ± 0.3/3.7 ± 0.4 (PVZ, SVZ, PZ)<br>6.9 ± 0.5/4.1 ± 0.4 (PVZ, SVZ)<br>2.1 ± 0.3/2 ± 0.2 (PVZ, SVZ, PZ)	+++<br>+++<br>+++
**Basal zone**	1 type, round<br>2 type, oval<br>Granules	4.9 ± 0.3/4.1 ± 0.5 (PVZ, SVZ)<br>–<br>2.2 ± 0.1/1.5 ± 0.3 (PVZ, SVZ, PZ)	+++<br>–<br>++	3.9 ± 0.5/3.7 ± 0.5 (PVZ, SVZ, PZ)<br>7.1 ± 0.6/5.7 ± 0.4 (PVZ, SVZ)<br>1.9 ± 0.3/1.9 ± 0.2 (PVZ, SVZ, PZ)	+++<br>+++<br>+++
**Granular Eminences**	1 type, round<br>Granules	4.2 ± 0.3/3.4 ± 0.1<br>2.4 ± 0.2/2 ± 0.3	+++<br>++	4.4 ± 0.5/3.3 ± 0.5<br>2.1 ± 0.4/2 ± 0.1	+++<br>++\+++

Optical density (OD) in cells was classified according to the following scale: high (220–170 UOD, which corresponds to +++) and moderate (170–120 UOD, which corresponds to ++). The large and small diameters of the cell body are shown with a slash (/). Cells moderately and intensely labeled are shown with a slash (\). PVZ - periventricular zone, SVZ - subventricular zone, PZ - parenchymal zone.

Table S3. Morphometric and densitometric characteristics of GS-labeled cells (M ± SD) in the intact and injured cerebellum of juvenile masu salmon, *Oncorhynchus masou*.

**Brain Areas**	**Intact Animals**			**Injured Cerebellum**	
	**GS**				
	**Type of cell**	**Cell Size, µm**	**Optical Density, UOD**	**Cell Size, µm**	**Optical Density, UOD**
**Dorsal zone**					
**Molecular layer**	1 type, round	4 ± 0.6/3.6 ± 0.2 (PVZ, SVZ, PZ)	++\+++	3.4 ± 0.3/3.1 ± 0.3 (PVZ, SVZ, PZ)	+++
	2 type, oval	6.3 ± 0.5/4.7 ± 0.5 (PVZ, SVZ)	++\+++	6.1 ± 0.6/4.6 ± 0.2 (PVZ, SVZ, PZ)	+++
	3 type, elongate	–	–	8.9 ± 1/5.5 ± 0.5 (PZ)	+++
	Radial glia round	4.4 ± 0.3/4 ± 0.2* (PVZ, SVZ, PZ)	+++	4.9 ± 0.3/4.2 ± 0.3* (PVZ, SVZ, PZ)	+++
	Radial glia oval	7.4 ± 0.7 /5.2 ± 0.4* (PVZ, SVZ, PZ)	+++	7.3 ± 0.5/5.3± 0.5* (PVZ, SVZ, PZ)	+++
**Ganglion layer**	1 type, round	4.2 ± 0.5/3.4 ± 0.4	+++	4.8 ± 0.3/4.1 ± 0.4	+++
	2 type, oval	6.5 ± 0.3/5.2 ± 0.4	+++	6.8 ± 0.6/55 ± 0.5	+++
	3 type, elongate	11.6 ± 0.7/6 ± 0.8	++\+++	10.3 ± 0.9/7.1 ± 0.6 (PZ)	+++
**Granular layer**	1 type, round	4.1 ± 0.5/3.6 ± 0.3	+++	5.4 ± 0.4/3.5 ± 0.5	+++
	2 type, oval	6.8± 0.6/4.7 ± 0.5	+++	7.2 ± 0.7/5.3 ± 0.3	+++
**Lateral zone**					
**Molecular layer**	1 type, round	4 ± 0.4/3.4 ± 0.5 (PVZ, SVZ, PZ)	++\+++	4.2 ± 0.3/3.6 ± 0.1 (PVZ, SVZ, PZ)	+++
	2 type, oval	6.5 ± 0.4/4.5 ± 0.2 (PVZ, SVZ)	++\+++	6.5 ± 0.4 /4.7 ± 0.3 (PVZ, SVZ, PZ)	+++
	3 type, elongate	–	–	8.5 ± 1 /6.6 ± 0.5 (PZ)	+++
	Radial glia round	4.6 ± 0.3/4 ± 0.3* (PVZ, SVZ, PZ)	++\+++	4.5 ± 0.3 /4 ± 0.3* (PVZ, SVZ)	+++
	Radial glia oval	6.9 ± 0.6/5.3 ± 0.3* (PVZ, SVZ, PZ)	+++	7.5 ± 0.5 /5.6 ± 0.6* (PVZ, SVZ)	+++
**Ganglion layer**	1 type, round	4.8 ± 0.5/4.1 ± 0.7	+++	5.2 ± 0.4/4.7 ± 0.7	+++
	2 type, oval	7.2 ± 0.5/5.1 ± 0.5	+++	6.8 ± 0.6/5.5 ± 0.5	+++
	3 type, elongate	11.6 ± 1/5.9 ± 0.6	++\+++	11.1 ± 1/5.9 ± 0.6	+++
**Granular layer**	1 type, round	4.4 ± 0.5/3.8 ± 0.3	+++	3.8 ± 0.2/3.6 ± 0.3	+++
	2 type, oval	6.7 ± 0.5/5.2 ± 0.5	+++	6 ± 0.5/4.8 ± 0.5	+++
**Basal zone**					
**Molecular layer**	1 type, round	4 ± 0.5/3.4 ± 0.5 (PVZ, SVZ, PZ)	++\+++	3.9 ± 0.5/3.3 ± 0.3 (PVZ, SVZ, PZ)	++\+++
	2 type, oval	6.2 ± 0.4/4.5 ± 0.5 (PVZ, SVZ)	++\+++	6.1 ± 0.6/5.1 ± 0.4 (PVZ, SVZ, PZ)	+++
	3 type, elongate	–	–	8.4 ± 0.6/5.9 ± 0.4 (PZ)	+++
	Radial glia round	5.3 ± 0.3/4.7 ± 0.2* (PVZ, SVZ, PZ)	+++	5.4 ± 0.4/4.9 ± 0.4* (PVZ, SVZ)	+++
	Radial glia oval	7 ± 0.6/5.4 ± 0.3*(PVZ, SVZ, PZ)	+++	7.1 ± 0.5/5.2 ± 0.3* (PVZ, SVZ)	+++
**Ganglion layer**	1 type, round	3.9 ± 0.5/3.4 ± 0.4	++\+++	4.3 ± 0.5/3.7 ± 0.7	++/+++
	2 type, oval	6.9 ± 0.5/5.1 ± 0.5	+++	7.1 ± 0.6/4.8 ± 0.5	+++
	3 type, elongate	11.9± 1.1/6.9 ± 0.5	++\+++	11.7 ± 1/6.8 ± 0.6	+++
**Granular layer**	1 type, round	4.3 ± 0.2/3.7 ± 0.4	++	4.9 ± 0.4/ 4.2 ± 0.4	+++
	2 type, oval	6.5 ± 0.7/4.6 ± 0.8	++	6.5 ± 0.6/4.8 ± 0.4	+++

Optical density (OD) in cells was classified according to the following scale: high (240–180 UOD, which corresponds to +++), moderate (180–120 UOD, which corresponds to ++). Large and small diameters of the cell body are shown through a forward slash (/). Cells moderately and intensely marked are shown with a slash (\). * - Radial glia. PVZ - periventricular zone, SVZ - subventricular zone, PZ - parenchymal zone.

Table S4. Morphometric and densitometric characteristics of CBS-marked cells (M ± SD) in the intact and injured cerebellum of juvenile masu salmon, *Oncorhynchus masou.*

**Brain Areas**	**Intact Animals**			**Injured Cerebellum**	
	**CBS**				
	**Type of cell**	**Cell Size, µm**	**Optical Density , UOD**	**Cell Size, µm**	**Optical Density , UOD**
**Dorsal zone**					
**Molecular layer**	1 type, round	4.5 ± 0.2/3.2 ± 0.3 (PVZ, SVZ, PZ)	++\+++	4.2 ± 0.4/3.4 ± 0.5 (PVZ, SVZ, PZ)	++\+++
	2 type, oval	7.6 ± 0.4/4.7 ± 0.4 (PVZ, SVZ, PZ)	+++	7.5 ± 0.5 /4.2 ± 0.4 (PVZ, SVZ, PZ)	+++

	3 type, elongate	–	–	9.8 ± 0.4/6.7 ± 0.5 (SVZ)	+++
**Ganglion layer**	1 type, round<br>2 type, oval	4.2 ± 0.2/3.3 ± 0.2<br>7.8 ± 0.4/5.6 ± 0.8	+++<br>+++	4.9 ± 0.3/4.3 ± 0.2<br>7.7 ± 0.3/5.5 ± 0.6	+++<br>+++
**Granular layer**	1 type, round<br>2 type, oval	4.1 ± 0.4/4 ± 0.6<br>7.1 ± 0.6/5.8 ± 0.4	++<br>++	4.4 ± 0.5/4 ± 0.7<br>6.9 ± 0.6/5.7 ± 0.5	++\+++<br>+++
**Lateral zone**					
**Molecular layer**	1 type, round<br>2 type, oval<br>3 type, elongate	4 ± 0.3/3.6 ± 0.2 (PVZ, SVZ, PZ)<br>7.2 ± 0.4/4.5 ± 0.4 (PVZ, SVZ, PZ)<br>–	++<br>++<br>–	4.1 ± 0.4/3.4 ± 0.3 (PVZ, SVZ, PZ)<br>6.9 ± 0.3/4.7± 0.3 (PVZ, SVZ, PZ)<br>9.5± 0.5/6.6 ± 0.4 (SVZ)	++\+++<br>++\+++<br>+++
**Ganglion layer**	1 type, round<br>2 type, oval<br>3 type, elongate	4.5 ± 0.4/3.5 ± 0.2<br>7.9 ± 0.6/5.5 ± 0.4<br>–	++<br>++<br>–	4.2 ± 0.2/4.2 ± 0.3<br>7.7 ± 0.9/5.4 ± 0.5<br>12.4 ± 1.1/7.1 ± 0.5	+++\++<br>++<br>++
**Granular layer**	1 type, round<br>2 type, oval	4.2 ± 0.3/4 ± 0.5<br>7.2 ± 0.3/5.7 ± 0.6	+<br>++	4.3 ± 0.3/3.7 ± 0.2<br>7.1 ± 0.7/5.2 ± 0.6	++\+++<br>++
**Basal zone**					
**Molecular layer**	1 type, round<br>2 type, oval<br>3 type, elongate	4.2 ± 0.4/3.4 ± 0.3 (PVZ, SVZ, PZ)<br>7.4 ± 0.5/5.7 ± 0.3 (PVZ, SVZ, PZ)<br>–	++<br>++<br>–	4.1 ± 0.3/3.4 ± 0.4 (PVZ, SVZ, PZ)<br>7.2 ± 0.5/4.7 ± 0.3 (PVZ, SVZ, PZ)<br>9.7 ± 1 /7.2 ± 0.7 (SVZ)	++\+++<br>++\+++<br>+++
**Ganglion layer**	1 type, round<br>2 type, oval<br>3 type, elongate	3.8 ± 0.4/3.9 ± 0.2<br>7.7 ± 0.5/4.6 ± 0.6<br>–	+++<br>+++<br>–	3.9 ± 0.3/4.1 ± 0.2<br>7.2 ± 0.5/4.9 ± 0.5<br>10.2 ± 0.9/6.8 ± 0.6	++\+++<br>++\+++<br>+++
**Granular layer**	1 type, round<br>2 type, oval	4.2 ± 0.4/4.3 ± 0.3<br>7.5 ± 0.4/6 ± 0.5	++<br>++	3.8 ± 0.5/ 4.5 ± 0.5<br>7.3± 0.5 /5.8 ± 0.2	++\+++<br>+++

Optical density (OD) in cells was classified according to the following scale: high (180–140 UOD, which corresponds to +++), moderate (140–90 UOD, which corresponds to ++) and less than 90 UOD, weak (corresponds to +). Large and small diameters of the cell body are shown through a forward slash (/). Cells moderately and intensely marked are shown with a slash (\). PVZ - periventricular zone, SVZ - subventricular zone, PZ - parenchymal zone.
